# Blastocyst-Derived Lactic Acid Regulates Uterine Epithelial Receptivity and Stromal Decidualization via the HIF1α-HO-1-Heme Metabolic Axis

**DOI:** 10.3390/antiox15091114

**Published:** 2026-09-04

**Authors:** Wen-Xu Yao, Yao-Dan Ma, Shi-Yao Ding, Jian Lu, Hao-Lan Tang, Zeng-Ming Yang

**Affiliations:** Key Laboratory of Animal Genetics, Breeding and Reproduction in the Plateau Mountain Region, College of Animal Science, Guizhou University, Guiyang 550025, China

**Keywords:** embryo implantation, lactic acid, HIF1α, HO-1, heme catabolism, bilirubin, epithelial receptivity

## Abstract

Successful embryo implantation requires intimate crosstalk between the blastocyst and the uterine epithelium within a defined window of receptivity. However, the metabolic signals that mediate this process in mammals remain poorly understood. In this study, pregnant mice, primary uterine cell culture and uterine epithelial organoids were used to examine the regulation and function of heme oxygenase-1 (HO-1) during mouse embryo implantation and decidualization. We demonstrate that embryo-derived lactic acid drives heme catabolism and regulates epithelial receptivity in mice through a hypoxia-inducible factor 1α (HIF1α) -heme oxygenase-1 (HO-1) signaling axis. Specifically, lactic acid stabilizes HIF1α to induce HO-1 expression in uterine epithelial cells by promoting von Hippel-Lindau (VHL) nucleolar sequestration and downregulating PHD2/3. Additionally, lactic acid suppresses the transcriptional repressor BACH1, further facilitating HO-1 induction. At physiological heme levels, HO-1-derived bilirubin promotes epithelial receptivity by increasing phosphorylated STAT3 (p-STAT3) and downregulating MUC1. A low dose of hemin promotes epithelial receptivity and decidualization, whereas a high dose of hemin suppresses these processes. Pharmacological inhibition of HO-1 in mice markedly reduces implantation sites, establishing the functional necessity of this pathway. However, when heme levels exceed the regulatory capacity of HO-1, epithelial dysfunction ensues, characterized by reduced p-STAT3 and elevated MUC1, which ultimately disrupts implantation. Consistent with this, chronic heme exposure by oral gavage in mice increases uterine heme levels and upregulates BACH1, thereby suppressing HO-1 and trapping the uterus in a non-receptive state, causing implantation failure. Our findings define a lactic acid-HIF1α-HO-1-heme metabolic checkpoint that couples glycolytic signaling to heme regulation and endometrial receptivity. Dysregulation of this checkpoint may contribute to implantation disorders associated with heme stress, providing mechanistic insights into heme stress-related uterine receptivity failure.

## 1. Introduction

Embryo implantation and decidualization are critical steps in pregnancy establishment across rodents and primates [1,2]. Disruption of these processes can lead to adverse outcomes, including recurrent implantation failure, recurrent pregnancy loss, and preeclampsia [3,4,5]. In mice and rats, endometrial receptivity is molecularly defined by upregulation of pro-receptive markers including phosphorylated STAT3 (p-STAT3) and amphiregulin (AREG), coupled with suppression of the anti-adhesive molecule MUC1 [6,7,8]. Although hormonal and transcriptional networks governing early pregnancy have been extensively characterized, embryo-derived metabolic signals that shape uterine receptivity remain poorly defined.

Sterile inflammation mediated by damage-associated molecular patterns (DAMPs) is integral to successful embryo implantation, but its untimely activation can compromise pregnancy outcomes in humans [9]. Previous studies have demonstrated that the release of sterile inflammatory mediators such as ATP and HMGB1 influences implantation and decidualization in mice [10,11]. Beyond these, heme is a potent pro-oxidative DAMP released from damaged vasculature and injured cells [12]. Heme is also a major component of dietary iron and is known to promote inflammation in the intestinal tract in mice [13,14]. Accordingly, dietary heme, such as that derived from red meat, is associated with increased colorectal cancer risk, as supported by studies in rats and mice [15,16]. More broadly, excess free heme induces epithelial dysfunction and amplifies sterile inflammation in multiple mucosal tissues in mice [15]. Free heme is also released during mouse in vitro decidualization [17,18]. The molecular machinery that safeguards the uterine epithelium against this heme-induced challenge remains poorly defined, and it is unclear whether an elevated heme burden can exceed the capacity of this protective system to impair fertility.

The primary means of degrading excess free heme is heme oxygenase-1 (HO-1), encoded by *Hmox1* [19]. HO-1 degrades free heme into biliverdin, carbon monoxide and ferrous iron in mice [20]. Biliverdin is subsequently converted to bilirubin, a potent antioxidant [21]. By degrading pro-oxidant free heme and generating antioxidant metabolites, HO-1 serves as a central regulator of cellular redox homeostasis [22]. Consistent with this protective function, HO-1 also mitigates heme-induced cytotoxicity in endometriosis lesions [23]. Beyond its role in endometriosis, HO-1 is critical for pregnancy maintenance. In mice, homozygous *Hmox1* deficiency leads to fetal death around gestational days 12–14, and heterozygous deficiency impairs placental and fetal growth from day 10 of pregnancy, highlighting the essential role of maternal HO-1 [24]. *Hmox1*-deficient mice also display aberrant intrauterine fetal growth restriction (IUGR) and fetal lethality [25,26]. Moreover, the molecular mechanisms by which HO-1 regulates embryo implantation during the peri-implantation period and the pathways through which free heme damages the placenta are poorly defined in mice [26,27].

To address these unknowns, we focused on the transcriptional regulation of *Hmox1*, which is known to be repressed by BTB and CNC homology (BACH) family factors [28]. BACH2 is largely restricted to immune cells, whereas BACH1 functions as the dominant heme-responsive repressor in epithelial tissues in humans [29,30]. Excess heme induces HO-1 expression and also reinforces BACH1-mediated repression of *Hmox1* in rats [31], suggesting that a dominant activating signal must overcome this repression to induce HO-1. The identity of this signal in the peri-implantation uterus remains unclear.

Hypoxia-inducible factor 1α (HIF1α) is a direct transcriptional activator of *Hmox1* [17] and a key candidate regulator of HO-1 during the peri-implantation period. On day 4 of pregnancy, the rat uterus becomes hypoxic (3–5% O_2_) as the blastocyst attaches to the uterine epithelium [32,33]. HIF1α is essential for embryonic tissue remodeling, as global deletion of *Hif1α* impairs vascular development and placentation in mice [34]. In addition to these functions in development, HIF1α regulates endometrial epithelial metabolism and redox homeostasis, both integral to uterine function during implantation [35,36]. In the mouse uterus, HIF1α is predominantly localized to the luminal epithelium during the peri-implantation period [37]. Under normal oxygen conditions, HIF1α is constitutively degraded via PHD-mediated hydroxylation and subsequent VHL-dependent ubiquitination [38,39]. Importantly, the hypoxic uterine niche drives a glycolytic shift that generates L-lactic acid (hereafter referred to as lactic acid), which further stabilizes HIF1α by inhibiting PHD2 activity in mice [40]. Thus, it remains unclear whether uterine hypoxia alone is sufficient to regulate heme homeostasis via HO-1, or whether additional embryo-derived signals are required for full activation of this pathway. Identifying these signals and defining how they activate the HIF1α and HO-1 pathway in the epithelium is essential for understanding implantation-associated heme homeostasis in mice.

Lactic acid, a product of aerobic glycolysis, remodels the uterine microenvironment and promotes local acidification [41,42,43]. Lactic acid enters uterine cells via monocarboxylate transporters (MCTs), where it stabilizes HIF1α through inhibition of prolyl hydroxylases, as demonstrated in mice [40], and lactylation of HIF1α across species, including mice and humans [44]. Consistently, Warburg-like glycolysis and a local lactic acid shuttle are activated in mouse decidua and are essential for early pregnancy [45], further supporting the importance of lactic acid signaling in the uterus. These findings raise the hypothesis that blastocyst-derived lactic acid acts as a paracrine signal to stabilize epithelial HIF1α, which then transcriptionally activates *Hmox1* to regulate heme, suppress oxidative damage, and establish endometrial receptivity. However, whether lactic acid actually regulates HIF1α through this HIF1α-HO-1 axis in mice remains to be fully investigated.

Here, we combine spatiotemporal expression profiling, pharmacological intervention, and dietary manipulation to delineate a lactic acid-HIF1α-HO-1-heme-regulatory axis in the mouse uterus. We demonstrate that lactic acid stabilizes epithelial HIF1α to induce HO-1 expression, which clears excess heme and maintains endometrial receptivity. Chronic dietary heme excess exceeds the regulatory capacity of this pathway. This leads to insufficient HO-1 induction, unrestrained heme accumulation, oxidative damage, and loss of uterine receptivity, ultimately resulting in implantation failure. Our findings define a previously unrecognized embryo-initiated metabolic checkpoint regulating maternal-embryonic crosstalk and provide mechanistic insights into metabolic stress-driven implantation disorders.

## 2. Materials and Methods

### 2.1. Animals and Treatments

Adult ICR mice (6–8 weeks old) were purchased from Hunan Jingda Laboratory Animal Co., LTD. All mice were maintained under controlled environmental conditions of 22 ± 1 °C with a 12 h light/12 h darkness cycle. All animal experimental procedures were approved by the Institutional Animal Use and Care Committee of Guizhou University (EAE-GZU-2023-T005) and were conducted in compliance with the National Research Council’s Guide for the Care and Use of Laboratory Animals. To generate pregnancy or pseudopregnancy mice, adult female mice were mated with fertile and vasectomized male mice, respectively. Detection of a vaginal plug was defined as day 1 (D1) of pregnancy or pseudopregnancy. On days 1–4, pregnancy was validated by embryo flushing from the oviducts or uteri. On day 5, implantation sites were confirmed via tail intravenous injection of 0.1 mL of 1% Chicago blue dye (C8679, Sigma-Aldrich, St. Louis, MO, USA). Uteri were collected at different days of pregnancy for further analyses. To further examine effects of lactic acid, a unilateral intrauterine instillation of 5 µL of 1 mM lactic acid (or saline as control) was performed in ovariectomized mice that had been allowed to recover for 2 weeks. Mice were sacrificed 3 h after the injection to collect uteri for further analysis.

### 2.2. Delayed Implantation and Activation

Delayed implantation and activation were performed as previously described [46]. Briefly, female mice were subjected to bilateral ovariectomy between 09:00 and 10:00 on day 4 of pregnancy. Mice received daily subcutaneous injections of progesterone (1 mg per mouse; E1024, Sigma-Aldrich, St. Louis, MO, USA) from days 5 to 7 to maintain delayed implantation. On day 7, implantation was activated by a single subcutaneous injection of 25 ng estradiol-17β (E8875, Sigma-Aldrich, St. Louis, MO, USA) in oil.

### 2.3. Intrauterine Injection of ZnPP

Intrauterine injection of zinc protoporphyrin IX (ZnPP, a competitive HO-1 inhibitor) was performed as previously described [10]. Briefly, ZnPP (HY-101193, MedChemExpress, Monmouth Junction, NJ, USA) was dissolved in 0.1 M NaOH, sonicated and heated to ensure complete dissolution, then diluted with sterile normal saline to 3 mg/mL, and adjusted to pH 7.4. All preparation steps were performed under light protection, and the solution was used within 2 h of preparation. At 10:00 on day 4 of pregnancy, a slow intrauterine injection of 3 µL ZnPP solution (3 mg/mL) was administered into one uterine horn of each mouse, while the contralateral horn received an equal volume of pH-matched vehicle (0.1 M NaOH in saline, pH 7.4) as an internal control.

### 2.4. Hemin-Induced Artificial Decidualization

Artificial decidualization was induced as previously described [47]. Briefly, hemin (H5128, Sigma-Aldrich) was dissolved in 0.1 M NaOH with sonication and mild heating, diluted to 10 mg/mL with sterile saline, adjusted to pH 7.4, and prepared fresh under light protection immediately before use. Vehicle consisted of saline containing an equivalent amount of NaOH, adjusted to pH 7.4. On day 4 of pseudopregnancy, one uterine horn of each mouse received an intraluminal injection of 10 µL hemin solution (10 mg/mL), while the contralateral horn received vehicle as an internal control. Mice were euthanized by cervical dislocation on day 8, and uterine were harvested for decidualization assessment.

### 2.5. Western Blot

Western blot was performed as previously described [48]. Tissues or cultured cells were lysed in RIPA buffer (R0010, Solarbio, Beijing, China), and protein concentration was determined using the BCA method (Thermo Fisher Scientific, Waltham, MA, USA). The protein samples were fractionated by 10% or 12% SDS-PAGE (polyacrylamide gel electrophoresis) and transferred onto PVDF membranes (IPVH00010, Millipore, Billerica, MA, USA). PVDF membranes were blocked with 5% non-fat milk (A600669, Sangon Biotech, Shanghai, China) for 1 h and then incubated with each primary antibody overnight at 4 °C. Primary antibodies used in this study included anti-GAPDH (1:1000, SC32233, Santa Cruz Biotechnology, Dallas, TX, USA), anti-HO-1 (1:1000, 10701-1-AP, Proteintech, Chicago, IL, USA), anti-HIF1α (1:1000, ab179483, Abcam, Cambridge, UK), anti-BACH1 (1:1000, 14018-1-AP, Proteintech, Chicago, IL, USA), anti-PHD2 (1:1000, 19886-1-AP, Proteintech, Chicago, IL, USA), anti-PHD3 (1:1000, 82377-1-RR, Proteintech, Chicago, IL, USA), anti-AREG (1:1000, bs-3847R, Bioss, Woburn, MA, USA), anti-p-STAT3 (1:1000, 9131S, Cell Signaling Technology, Danvers, MA, USA), anti-MUC1 (1:1000, ab45167, Abcam, Cambridge, UK), anti-VHL (1:1000, GTX101087, GeneTex, Irvine, CA, USA), anti-BACH2 (1:1000, ab320721, Abcam, Cambridge, UK), anti-HO-2 (1:1000, 14817-1-AP, Proteintech, Chicago, IL, USA), and anti-α-Tubulin (1:1000, 2144S, Cell Signaling Technology, Danvers, MA, USA). After being incubated with primary antibodies, membranes were washed three times with TBST (10 min each). Subsequently, membranes were incubated with HRP-conjugated secondary antibody (1:5000, Invitrogen, Carlsbad, CA, USA) for 1 h at room temperature. After additional washes, signals were detected using the Chemiluminescent HRP Substrate kit (Millipore, Billerica, MA, USA).

### 2.6. RNA Extraction and Quantitative Real-Time PCR

qPCR was performed as previously described [49]. Total RNAs were extracted from mouse uterine stromal cells using TRIzol reagent (9109, Takara, Kusatsu, Japan). cDNA was synthesized from RNA with the HiScript II Q RT SuperMix kit (R222-01-AB, Vazyme, Nanjing, China). qPCR was carried out with SYBR Premix (Q311-02-AA, Vazyme, Nanjing, China). Relative expression was determined by the 2^−△△^ CT method and normalized to RPL7. Primer sequences were provided in Table 1.

### 2.7. Immunofluorescence

Immunofluorescence was performed as previously described [50]. Briefly, paraffin sections, frozen sections, or cultured cells were blocked with 10% horse serum for 1 h at 37 °C, and then incubated with each primary antibody overnight at 4 °C in a humid chamber. Primary antibodies used were as follows: anti-HO-1 (1:200, 10701-1-AP, Proteintech, Chicago, IL, USA), anti-HIF1α (1:200, ab179483, Abcam, Cambridge, UK), anti-BACH1 (1:200, 14018-1-AP, Proteintech, Chicago, IL, USA), anti-PHD2 (1:200, 19886-1-AP, Proteintech, Chicago, IL, USA), anti-PHD3 (1:200, 82377-1-RR, Proteintech, Chicago, IL, USA), anti-LDHA (1:200, 3582S, Cell Signaling Technology, Danvers, MA, USA), anti-ATP6V1B2 (1:200, 15097-1-AP, Proteintech, Chicago, IL, USA), anti-HCAR2 (1:200, A15611, ABclonal, Woburn, MA, USA), anti-GPR81 (1:200, 20146-1-AP, Proteintech, Chicago, IL, USA), anti-HO-2 (1:200, 14817-1-AP, Proteintech, Chicago, IL, USA), anti-NCL (1:500, 14574s, Cell Signaling Technology, Danvers, MA, USA), anti-MUC1 (1:200, ab45167, Abcam, Cambridge, UK), and anti-p-STAT3 (1:200, 9131S, Cell Signaling Technology, Danvers, MA, USA). Paraffin sections, frozen sections, or cultured cells were then incubated with Alexa 488-conjugated secondary antibodies. Nuclei were counterstained with propidium iodide (PI, Sigma-Aldrich) or DAPI (D9542, Merck, Rahway, NJ, USA) for 35 min at 37 °C. Fluorescence signals were acquired by using Nikon ECLIPSE Ti2 confocal microscope (Nikon Corp., Tokyo, Japan).

### 2.8. Tyramide Signal Amplification (TSA) Multiplex Immunofluorescence

TSA multiplex immunofluorescence was performed according to the manufacturer’s instructions (Bry-880488, Shanghai Ruiyu Biotechnology, Shanghai, China). Briefly, paraffin sections were dewaxed, rehydrated, and subjected to antigen retrieval in EDTA buffer (pH 9.0). After cooling, sections were incubated with hydrogen peroxide blocking solution for 10 min and washed three times with PBS (5 min each). Cultured cells were fixed and permeabilized as required. All samples were then blocked with 10% horse serum for 1 h at 37 °C and incubated with primary antibodies overnight at 4 °C in a humidified chamber. Subsequently, samples were incubated with HRP-conjugated secondary antibodies for 30 min at 37 °C, followed by CF488 Tyramide reagent for 10 min according to the kit protocol. After three PBS washes, the first staining cycle was completed. For subsequent cycles, paraffin sections underwent microwave retrieval in citrate buffer (pH 6.0) for 10 min and cooled to room temperature. Cultured cells were incubated with Antibody Eluent (abs994, Absin, Shanghai, China) for 20 min at 37 °C, washed with PBS, and processed for the next cycle. Primary antibodies used were as follows: anti-ATP6V1A (1:500, 17115-1-AP, Proteintech, Chicago, IL, USA), anti-VHL (1:500, GTX101087, GeneTex, Irvine, CA, USA). Nuclei were counterstained with DAPI and imaged by confocal microscope.

### 2.9. Isolation and Treatment of Mouse Endometrial Epithelial Cells

Mouse endometrial epithelial cells were isolated as previously described [51]. Briefly, Uteri from day 4 pseudopregnancy mice were opened longitudinally, washed in HBSS (H4891, Sigma-Aldrich), and sequentially digested in HBSS with 0.2% trypsin (0458, Amresco, Solon, OH, USA) and 6 mg/mL dispase II (04942078001, Roche Applied Science, Basel, Switzerland) for 1.5 h at 4 °C, 30 min at room temperature and 10 min at 37 °C. Following HBSS washes, luminal epithelial cells were harvested and maintained in DMEM/F12 (D2906, Sigma-Aldrich, St. Louis, MO, USA) supplemented with 10% charcoal-treated FBS (040011A, Biological Industries, Cromwell, CT, USA). Ultroser G (15950-017, Sartorius, Göttingen, Germany) served as a serum substitute for FBS in animal cell culture. Cells were cultured in DMEM/F12 supplemented with 1% Ultroser G for 12 h before treatment. The reagents used for epithelial cells treatments included lactic acid (L118493, Aladdin, Shanghai, China), HPPE (HY-153040, MedChemExpress, Monmouth Junction, NJ, USA), Hemin (51280, Sigma-Aldrich, St. Louis, MO, USA), 5-Aminolevulinic acid (ALA; HY-W000450, MedChemExpress, Monmouth Junction, NJ, USA), Bilirubin (14370, Sigma-Aldrich, St. Louis, MO, USA), CoCl_2_ (C8661, Sigma-Aldrich, St. Louis, MO, USA), ZnPP (HY-101193, MedChemExpress, Monmouth Junction, NJ, USA), mouse TNF (410-MT-010, Bio-Techne, Minneapolis, MN, USA), and mouse CTSB (50084-M08H, Sino Biological, Beijing, China).

### 2.10. Isolation, Culture, and Decidualization of Uterine Stromal Cells

Mouse endometrial stromal cells were isolated as previously described [52]. Briefly, uteri on day 4 of pseudopregnancy were rinsed with HBSS and then digested in 3.5 mL HBSS supplemented with 1% (*w*/*v*) trypsin and 6 mg/mL dispase II. After removal of luminal epithelial cells by HBSS washing, the remaining tissues were further digested in 6 mL HBSS containing 0.15 mg/mL collagenase I (17100-017, Invitrogen, Houston, TX, USA) for 35 min at 37 °C. The stromal cells were harvested, plated and cultured in DMEM/F12 supplemented with 2% charcoal-treated FBS (cFBS, Biological Industries, Cromwell, CT, USA). After 2 h, the medium containing unattached epithelial cells was aspirated and replaced with DMEM/F12 containing 10% heat-inactivated FBS for continued stromal cell expansion. To induce in vitro decidualization, stromal cells were cultured with 10 nM estradiol-17β (E) and 1 µM progesterone (P) in DMEM/F12 supplemented with 2% cFBS. The reagents used for cell treatments included Hemin (51280, Sigma-Aldrich, St. Louis, MO, USA), 5-Aminolevulinic acid (ALA; HY-W000450, MedChemExpress, Monmouth Junction, NJ, USA), and Bilirubin (14370, Sigma-Aldrich, St. Louis, MO, USA).

### 2.11. Culture and Treatment of Endometrial Epithelial Organoids

Uterine luminal epithelial organoids were isolated and cultured as previously described [49]. Briefly, uteri on day 4 of pseudopregnancy were dissected longitudinally, rinsed with HBSS, and digested at 4 °C for 1 h, room temperature for 1 h, and 37 °C for 8 min. After digestion, the epithelial cells were rinsed with HBSS, collected, and pelleted by centrifugation. After removing the supernatant, cell pellet was resuspended in DMEM/F12 to a density of 1.5 × 10^7^ cells/mL. The cell suspension was mixed with ice-cold ECM (1:3, 356231, Corning, Bedford, MA, USA) and seeded onto the preheated 24-well plates. Cultures were maintained at 37 °C in a CO_2_ incubator with organoid medium. The organoids with lumen were formed within 5–7 days culture. The reagents used for organoids treatments included lactic acid (L118493, Aladdin, Shanghai, China), Hemin (51280, Sigma-Aldrich, St. Louis, MO, USA), Bilirubin (14370, Sigma-Aldrich, St. Louis, MO, USA), and ZnPP (HY-101193, MedChemExpress, Monmouth Junction, NJ, USA).

### 2.12. LysoSensor Green DND-189 Staining

LysoSensor Green DND-189 staining was performed as previously described [53]. Briefly, approximately 20 min before each time point, 3 µL of LysoSensor Green DND-189 (3 µM; L7535, Invitrogen, Carlsbad, CA, USA) was injected into both uterine horns of day 4 pregnant or pseudopregnant mice. Uteri were collected at 08:00, 12:00, 16:00, and 20:00 on day 4 of pregnancy, and at 20:00 on day 4 of pseudopregnancy, and immediately snap-frozen in liquid nitrogen. Frozen sections were cut, heated at 55 °C for 20 min, counterstained with DAPI, and imaged under a fluorescence microscope with excitation at 488 nm.

### 2.13. Colorimetric Assay for Lactic Acid

Uterine luminal fluid was collected by flushing the uterine lumen with 100 µL of saline on day 4 of pregnancy or pseudopregnancy and centrifuged at 3000 rpm for 20 min to remove cellular debris and embryos. Uterine tissues on day 4 of pregnancy or pseudopregnancy were homogenized in PBS (0.01 M, pH 7.4) containing 1 mM PMSF and centrifuged at 10,000× *g* for 10 min at 4 °C. The supernatants were collected and kept on ice. Protein concentrations were determined using a BCA Protein Assay Kit (23225, Thermo Fisher Scientific, Waltham, MA, USA) and equalized across samples. Lactic acid levels were then measured using a lactic acid colorimetric assay kit (E-BC-K044-M, Elabscience, Wuhan, China) according to the manufacturer’s instructions, and normalized to protein content.

### 2.14. Enzyme-Linked Immunosorbent Assay (ELISA)

Uterine luminal fluid and tissue homogenates were prepared as described above. LDH levels in uterine homogenates from day 4 pregnant or pseudopregnant mice were measured using a mouse LDH ELISA kit (ELK2242, ELK Biotechnology, Wuhan, China). Hemoglobin (Hb) levels in uterine luminal fluid from day 4 pregnant or pseudopregnant mice, and ovariectomized mice were quantified using a mouse hemoglobin ELISA kit (BY-EM220745, Boyan Biotech, Shanghai, China). Heme levels were measured in the following samples using a mouse heme ELISA kit (MM-44917M1, Meimian Biotech, Yancheng, China): uterine luminal fluid from day 4 pregnant mice, day 4 pseudopregnant mice and ovariectomized mice. Hemin levels were also measured in uterine homogenates from day 4 pregnant mie, day 4 pseudopregnant mice, day 5 implantation sites (IS) and non-implantation sites (NS), hemin-treated mice on day 4, and cell culture supernatants. All assays were performed according to the manufacturer’s instructions.

### 2.15. Data Collection and Promoter Analysis

Promoter prediction and data collection were performed as previously described [54]. Briefly, bulk RNA-sequencing (RNA-seq) datasets for *Hmox1* (encoding HO-1) were obtained from the Gene Expression Omnibus (GEO) database (https://www.ncbi.nlm.nih.gov/gene/15368) (accessed on 23 July 2026). To explore the regulatory relationship between HIF1α and its target gene *Hmox1*, transcription factor binding sites (TFBS) within the *Hmox1* promoter were predicted using the JASPAR 2026 database (https://jaspar.elixir.no/).

### 2.16. Hemin Gavage in Mice

The hemin mouse model was established as previously described [15]. Briefly, hemin (H51280, Sigma-Aldrich, St. Louis, MO, USA) was dissolved in 0.1 M NaOH, sonicated with mild heating to ensure complete dissolution, then diluted with sterile normal saline to the 10 mg/mL working concentration and adjusted to pH7.4. The solution was protected from light throughout preparation and used within 2 h. Female mice (body weight ~25 g) received 0.2 mL of 10 mg/mL hemin (80 mg/kg/d body weight) or an equivalent volume of vehicle by daily oral gavage for 28 consecutive days. Treated females were then paired with males for mating, and daily hemin gavage was continued until mice were sacrificed on days 4, 5, or 8 of pregnancy. Uteri were collected for further analyses.

### 2.17. Statistical Analysis

All experiments were performed with at least three independent biological replicates. *n* denotes the number of biological replicates (separate animals or independent cell/organoid isolations). Data were presented as the mean ± standard deviation (SD) unless otherwise specified. The difference between groups was evaluated by one-way ANOVA followed by a two-tailed unpaired Student’s *t*-test. Statistical significance was defined as *: *p* < 0.05; **: *p* < 0.01; ***: *p* < 0.001.

## 3. Results

### 3.1. Embryonic Signals Selectively Induce Uterine HO-1 in the Peri-Implantation Mouse Uterus

**Figure 1:**
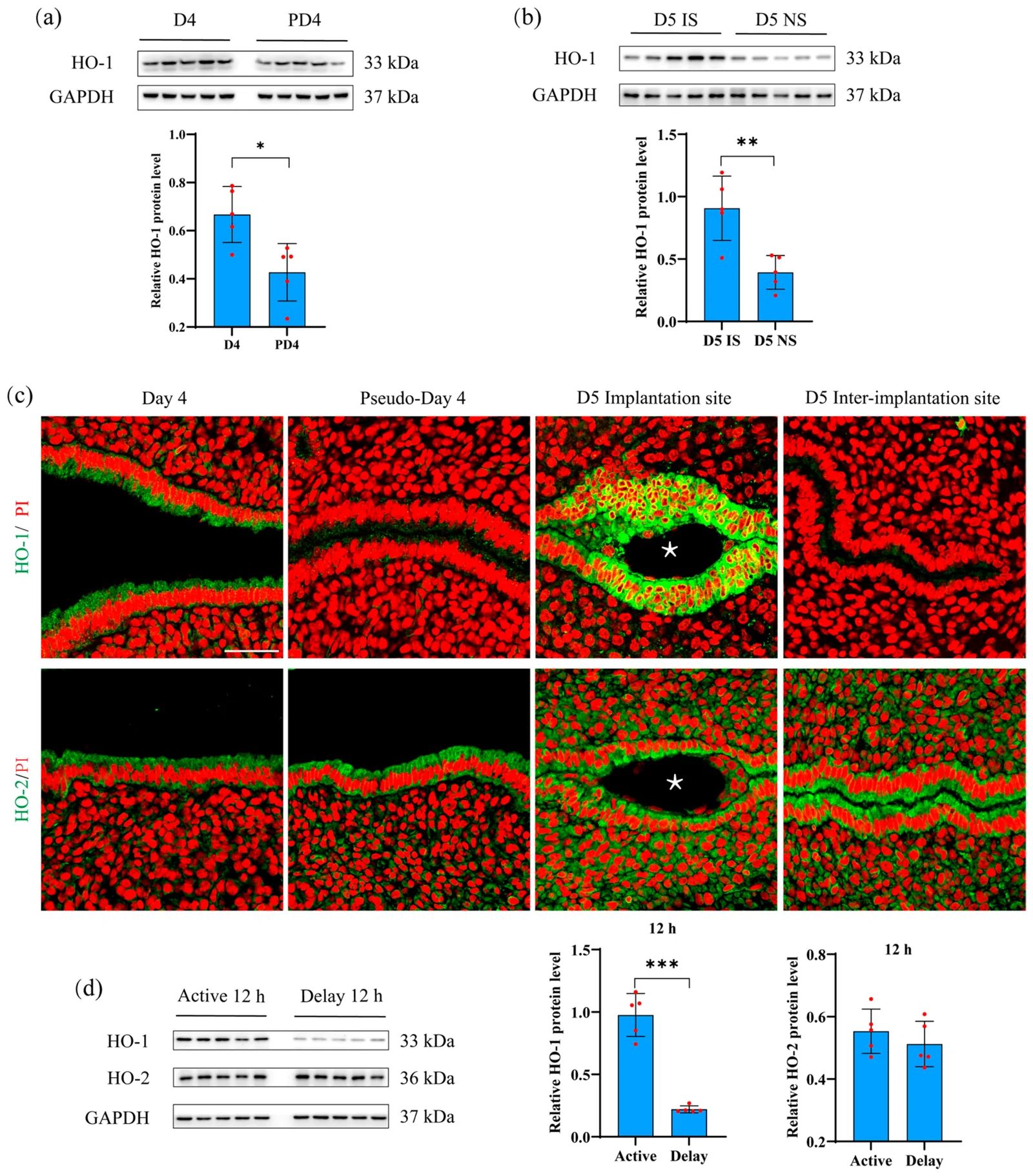
Effects of embryonic signals on uterine HO-1 expression in the peri-implantation mouse uterus. (**a**) Western blot analysis and quantification of HO-1 in mouse uteri on day 4 of pregnancy (D4) and day 4 of pseudopregnancy (PD4) (*n* = 5 per group). Red dot in histogram refers to one biological replicate. (**b**) Western blot analysis of HO-1 protein levels in mouse uteri at implantation sites (IS) and non-implantation sites (NS) on day 5 of pregnancy (D5) (*n* = 5 per group). (**c**) Immunofluorescence of HO-1 and HO-2 in mouse uteri on day 4 of pregnancy and day 4 of pseudopregnancy, at implantation sites and inter-implantation sites on day 5. Nuclei were counterstained with PI (red). Scale bar = 50 µm. ☆, Embryo. (**d**) Western blot analysis of HO-1 and HO-2 protein levels in mouse uteri under delayed implantation (*n* = 5 per group) and 12 h after delayed implantation was activated by estrogen (*n* = 5 per group). Data were presented as mean ± SD. *: *p* < 0.05; **: *p* < 0.01; ***: *p* < 0.001, by two-tailed Student’s *t*-test.

Heme oxygenase (HO) catalyzes the rate-limiting step in heme degradation and comprises two functionally distinct isoforms, the stress-responsive HO-1, which serves as the primary cellular regulator of heme homeostasis, and the constitutively active HO-2 [55,56]. We first compared uterine HO-1 expression in pregnant and pseudopregnant mice by Western blot. Compared with day 4 of pseudopregnancy, uterine HO-1 protein levels were significantly higher on day 4 of pregnancy (Figure 1a). On day 5 of pregnancy, the protein levels of HO-1 at implantation sites were significantly higher than that those at inter-implantation sites (Figure 1b). Immunofluorescence revealed that, compared with day 4 of pseudopregnancy, HO-1 was predominantly localized to the luminal epithelium on day 4 of pregnancy. On day 5, strong HO-1 immunofluorescence was detected in the luminal epithelium at implantation sites, whereas only weak signals were observed in the inter-implantation epithelium (Figure 1c). For HO-2 immunofluorescence, compared with day 4 of pregnancy, HO-2 fluorescence intensity was higher in the luminal epithelium of pseudopregnant uteri on day 4. On day 5, inter-implantation sites exhibited modestly stronger HO-2 signals than implantation sites (Figure 1c).

Together, these data indicate that the embryo should be involved in inducing HO-1 in the peri-implantation uterus. To further verify this, we employed a delayed implantation model, in which dormant blastocysts are activated by estrogen. HO-1 protein abundance was significantly increased 12 h after delayed implantation was activated by estrogen, whereas uterine HO-2 levels remained unchanged (Figure 1d). Collectively, these results suggest that the blastocyst is involved in the regulation of HO-1 in the mouse uterus during early pregnancy. We therefore focused subsequent analyses on HO-1 and its upstream regulation by embryo-derived signals.

### 3.2. Embryo-Derived Lactic Acid Induces Uterine Epithelial HO-1 and Participates in Luminal Acidification During the Peri-Implantation Period

**Figure 2:**
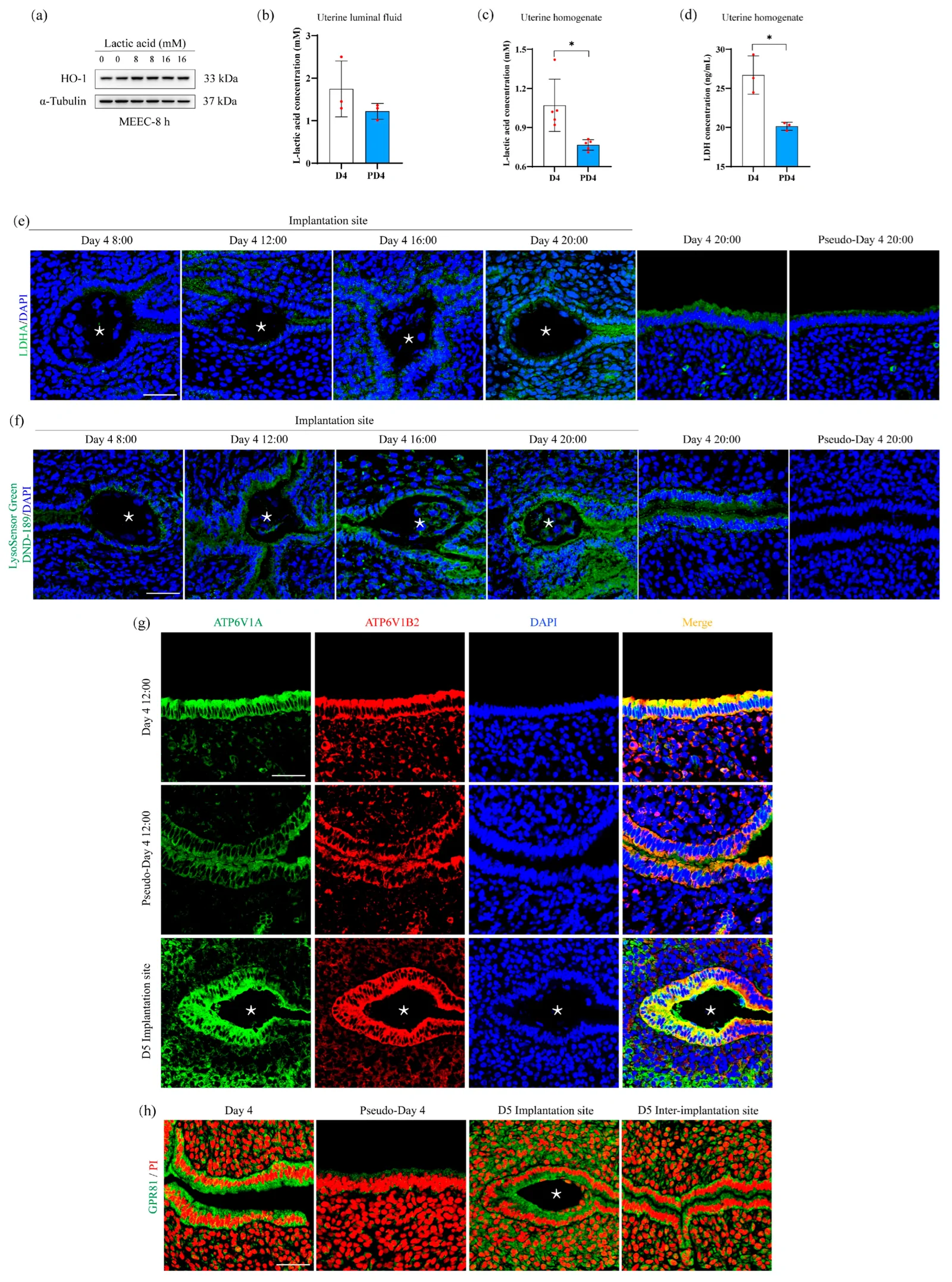
Embryo-derived lactic acid induces uterine epithelial HO-1 and uterine acidification during the peri-implantation period. (**a**) Western blot of HO-1 protein levels after endometrial epithelial cells were treated with different concentrations of lactic acid for 8 h. (**b**) Lactic acid concentration in uterine luminal fluid from day 4 pregnant (D4) and day 4 pseudopregnant (PD4) mice (*n* = 3 per group). (**c**) Lactic acid concentration in uterine homogenates from day 4 pregnant (D4) and day 4 pseudopregnant (PD4) mice (*n* = 5 per group). (**d**) Lactic acid dehydrogenase (LDH) concentration in uterine homogenates from day 4 pregnant (D4) and day 4 pseudopregnant (PD4) mice (*n* = 3 per group). (**e**) Immunofluorescence for LDHA in uterine sections from mice on day 4 of pregnancy at 08:00, 12:00, 16:00 and 20:00, and on day 4 of pseudopregnancy at 20:00. Nuclei were counterstained with DAPI (blue). Scale bar = 50 µm. ☆ Embryo. (**f**) Fluorescence of LysoSensor Green DND-189 in mouse uteri on day 4 of pregnancy at 08:00, 12:00, 16:00 and 20:00 and day 4 of pseudopregnancy. Scale bar = 50 µm. ☆ Embryo. (**g**) TSA immunofluorescence of ATP6V1A and ATP6V1B2 in mouse uteri on day 4 of pregnancy and day 4 of pseudopregnancy, at implantation sites and inter-implantation sites on day 5. Scale bar = 50 µm. ☆ Embryo. (**h**) Immunofluorescence of GPR81 in mouse uteri on day 4 of pregnancy and day 4 of pseudopregnancy, at implantation sites and inter-implantation sites on day 5. Scale bar = 50 µm. ☆ Embryo. Data were presented as mean ± SD. *: *p* < 0.05, by two-tailed Student’s *t*-test.

Considering that the embryo contributes to the regulation of uterine HO-1, we sought to identify how the embryo-derived molecules drive HO-1 induction in the uterus. The implanting blastocyst secretes a variety of paracrine factors, including tumor necrosis factor-α (TNF), cathepsin B (CTSB), and lactic acid [42,57,58,59]. We previously showed that the concentration of lactic acid in embryo culture medium increases significantly after 48 h of culture [60], demonstrating that the blastocyst actively secretes lactic acid. Treatment with recombinant TNF or CTSB did not significantly alter HO-1 protein levels in mouse endometrial epithelial cells (mEECs) compared with untreated controls (Figure S1a,b, Supporting Information). In contrast, treatment of mEECs with lactic acid increased HO-1 protein expression (Figure 2a), indicating that lactic acid is sufficient to induce HO-1 in uterine epithelial cells.

Having shown that lactic acid can regulate HO-1, we next asked whether lactic acid accumulates in the peri-implantation uterus. We measured lactic acid levels in uterine luminal fluid and tissue homogenates by lactic acid colorimetric assay kit. Lactic acid levels in uterine luminal fluid tended to be higher on day 4 of pregnancy than on day 4 of pseudopregnancy, although the difference was not statistical significance (Figure 2b). We next measured lactic acid levels in uterine tissue homogenates. Lactic acid levels were significantly higher in uterine homogenates from pregnant mice than from pseudopregnant mice on day 4 (Figure 2c). Consistent with this, we observed a marked increase in total LDH protein in uterine homogenates on day 4 of pregnancy (Figure 2d). Because elevated total LDH protein in uterine homogenates could result from non-specific leakage of intracellular contents, we next examined the expression and localization of LDHA, the rate-limiting enzyme that converts pyruvate to lactic acid [61]. LDHA immunofluorescence in the peri-embryonic luminal epithelium remained at a moderate level from 08:00 to 16:00 on day 4 of pregnancy, but increased markedly by 20:00, reaching intensities substantially higher than those in time-matched pseudopregnant uteri (Figure 2e). LDHA was also detectable in pseudopregnant uteri. This observation indicates that the uterine epithelium has an intrinsic capacity for lactic acid production. Nevertheless, LDHA expression in pseudopregnant uteri was consistently lower than in pregnant uteri at the corresponding time points. Together, these findings suggest that the uterus can produce lactic acid on its own. The presence of the embryo significantly amplifies this production. Embryonic signals are therefore the key trigger for the robust accumulation of lactic acid in the uterus during the peri implantation period.

To determine whether acidification occurs in the uterus, we used the acidotropic probe LysoSensor Green DND-189 (pKa ~ 5.2) [53,62], which accumulates in acidic compartments. We stained longitudinal uterine sections with LysoSensor Green DND-189 at 8:00, 12:00, 16:00 and 20:00 on day 4 of pregnancy. The fluorescence signal increased progressively and reached its highest intensity at 20:00 (Figure 2f). When we compared pregnant and pseudopregnant uteri at this peak time point, LysoSensor Green fluorescence in the luminal epithelium was substantially higher in pregnant uteri than in pseudopregnant uteri (Figure 2f). These results show that embryo-derived signals drive progressive acidification of the luminal epithelium at the implantation site.

To investigate the mechanism underlying this embryo-dependent acidification, we examined the expression of two core subunits of the vacuolar-type ATPase (V-ATPase), ATP6V1A and ATP6V1B2. The V-ATPase is a multi-subunit complex that drives ATP-dependent proton transport to acidify intracellular compartments and mediate transepithelial H^+^ secretion [63]. ATP6V1A and ATP6V1B2 thus function as the principal enzymatic executors of V-ATPase-mediated acidification, operating both at the plasma membrane and on intracellular organelles [64]. Immunofluorescence showed that, compared with day 4 pseudopregnant uteri, ATP6V1A and ATP6V1B2 were markedly increased in the uterine epithelium of day 4 pregnant uteri (Figure 2g). On day 5, these subunits were predominantly detected in the luminal epithelium (Figure 2g).

To explore how lactic acid signals in this acidic niche, we next examined the expression of its receptor GPR81 (encoded by *Gpr81*), a G protein-coupled receptor that senses lactic acid [65]. Compared with day 4 pseudopregnant uteri, GPR81 was markedly upregulated in the luminal epithelium of day 4 pregnant uteri (Figure 2h). On day 5, GPR81 immunofluorescence was stronger in the luminal epithelium at implantation sites than in inter-implantation regions (Figure 2h).

### 3.3. Peri-Implantation Hypoxia Induces Epithelial HO-1 via HIF1α in the Mouse Uterus

**Figure 3:**
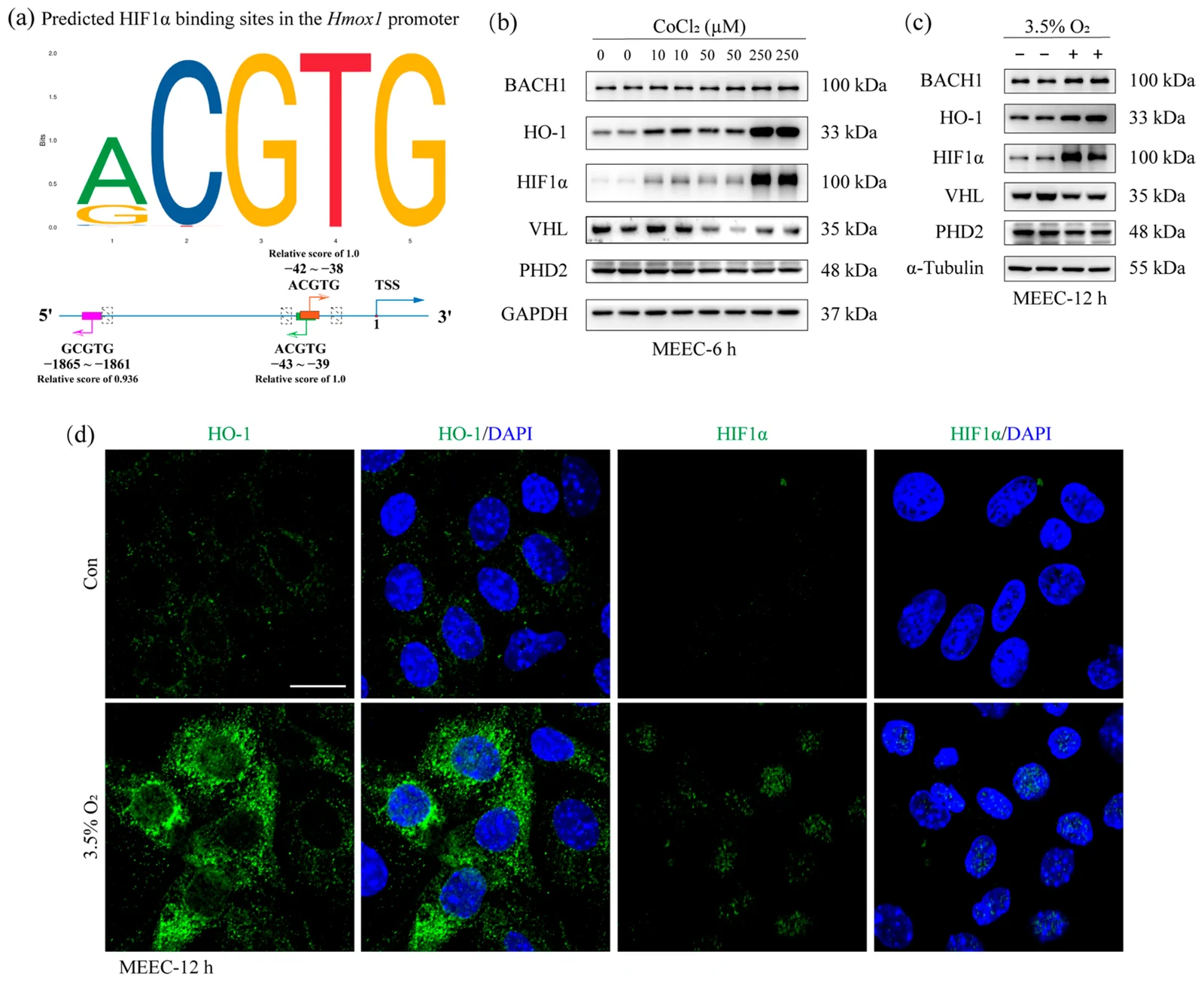
Peri-implantation hypoxia induces epithelial HO-1 via HIF1α in the mouse uterus. (**a**) Predicted HIF1α binding sites in the *Hmox1* promoter. Top, sequence logo of the HIF1α motif (MA0259.2). Bottom, schematic of the *Hmox1* promoter (−2000 to +100 bp from the TSS) showing three putative HIF1α binding sites. Rightward arrows indicate sense strand sites; leftward arrows indicate antisense strand sites. TSS, transcription start site. (**b**) Western blot analysis of BACH1, HO-1, HIF1α, PHD2, and VHL protein levels after endometrial epithelial cells were treated with different concentrations of CoCl_2_ for 6 h. (**c**) Western blot analysis of BACH1, HO-1, HIF1α, PHD2, and VHL protein levels after endometrial epithelial cells were treated with 3.5% O_2_ for 12 h. (**d**) Immunofluorescence analysis of HO-1 and HIF1α in endometrial epithelial cells treated with 3.5% O_2_ for 12 h. Scale bar = 150 µm.

Our data showed that HO-1 was detected in the luminal epithelium on both day 4 of pregnancy and day 4 of pseudopregnancy although the levels on day 4 of pregnancy were significantly higher than those on day 4 of pseudopregnancy. Given that the peri-implantation uterus is known to be hypoxic and HIF1α is localized to the luminal epithelium [34,37], we explored whether HIF1α induces HO-1 expression in luminal epithelium. We confirmed HIF1α localization in the peri-implantation uterus by immunofluorescence (Figure S2, Supporting Information). To test whether HIF1α might regulate *Hmox1*, we analyzed the mouse *Hmox1* promoter for potential HIF1α-binding sites. Using the JASPAR database [54], we scanned the region −2000 to +100 bp from the transcription start site and identified three putative HIF1α-binding sites with relative scores > 0.85 (Figure 3a). Thus, the *Hmox1* promoter contains three predicted HIF1α-binding sites, suggesting that HIF1α may directly regulate *Hmox1* transcription.

To test this possibility, we first asked whether HIF1α stabilization is sufficient to induce HO-1. We treated mEECs with the hypoxia mimetic CoCl_2_ [66]. CoCl_2_ (250 µM, 6 h) increased HIF1α protein levels, and this was accompanied by a concomitant upregulation of HO-1 (Figure 3b), demonstrating that chemical stabilization of HIF1α can drive HO-1 expression. We next examined whether the physiological hypoxia of the peri-implantation uterus could similarly activate this pathway. We cultured mEECs under a physiologically relevant low-oxygen condition (3.5% O_2_). Direct hypoxic exposure similarly elevated both HIF1α and HO-1 protein levels (Figure 3c). Immunofluorescence analysis confirmed enhanced nuclear HIF1α and cytoplasmic HO-1 signals under 3.5% O_2_ (Figure 3d). Collectively, these results indicate that the hypoxic environment of the peri-implantation uterus is capable of engaging the HIF1α pathway to support HO-1 expression.

### 3.4. Lactic Acid Stabilizes HIF1α Through PHD2/3 Downregulation and VHL Nucleolar Sequestration, and Suppresses BACH1 to Drive Uterine Epithelial HO-1 Expression

Having demonstrated that hypoxia can induce HO-1, we next investigated how lactic acid enhances HIF1α stability to upregulate HO-1 expression. We examined whether lactic acid affects HIF1α stability through the canonical prolyl hydroxylation pathway. In this pathway, prolyl hydroxylases (PHDs) hydroxylate HIF1α at conserved proline residues, targeting it for VHL-mediated proteasomal degradation [38,67]. Of the three PHD isoforms, PHD1 is predominantly nuclear and preferentially hydroxylates HIF2α [68]. PHD2 serves as the principal HIF1α prolyl hydroxylase, displaying the highest catalytic activity toward its key residues, while PHD3 can target both HIF1α and HIF2α [68,69]. We therefore focused on PHD2 and PHD3 and assessed whether lactic acid interferes with this hydroxylation step.

**Figure 4:**
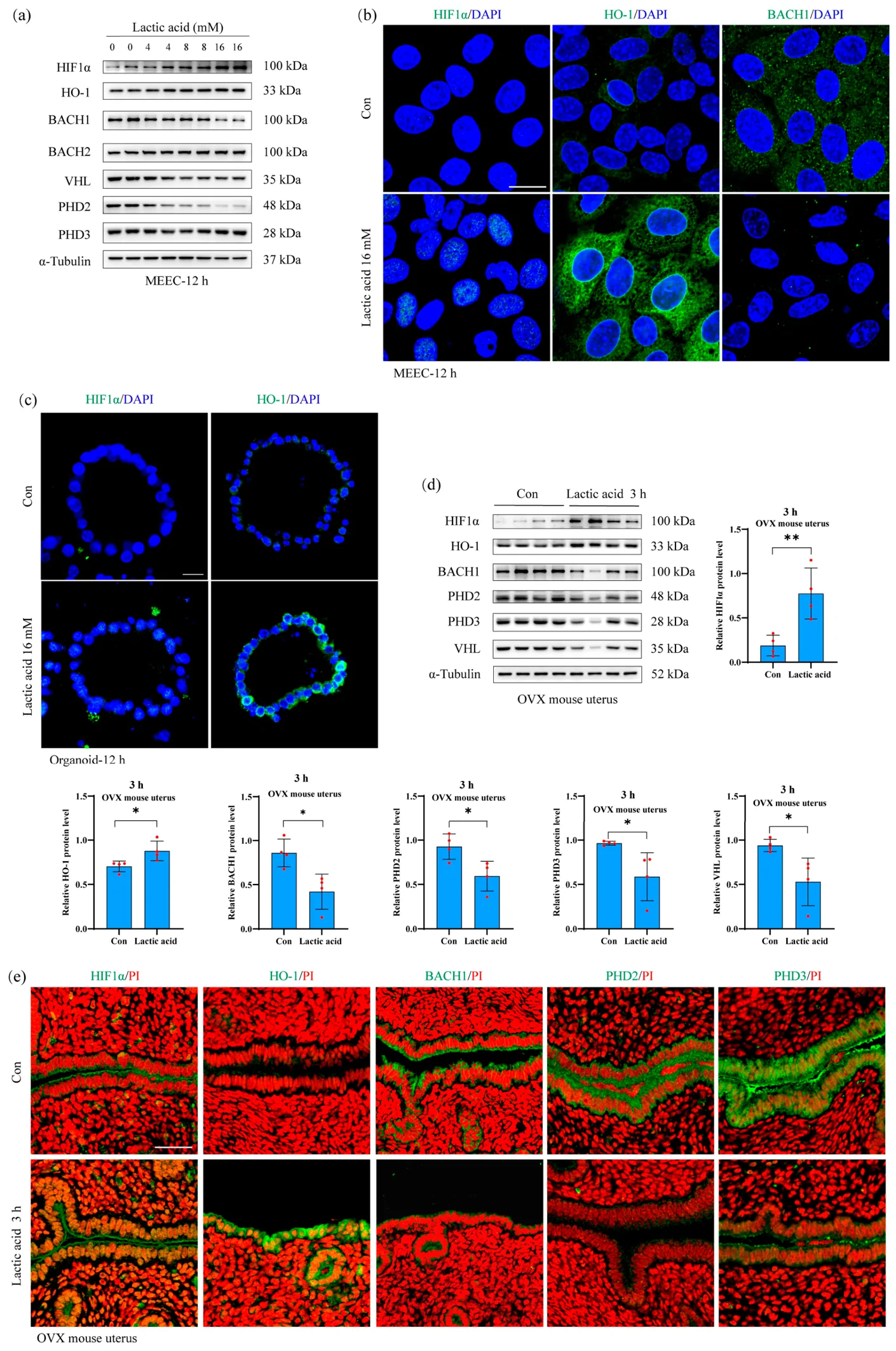

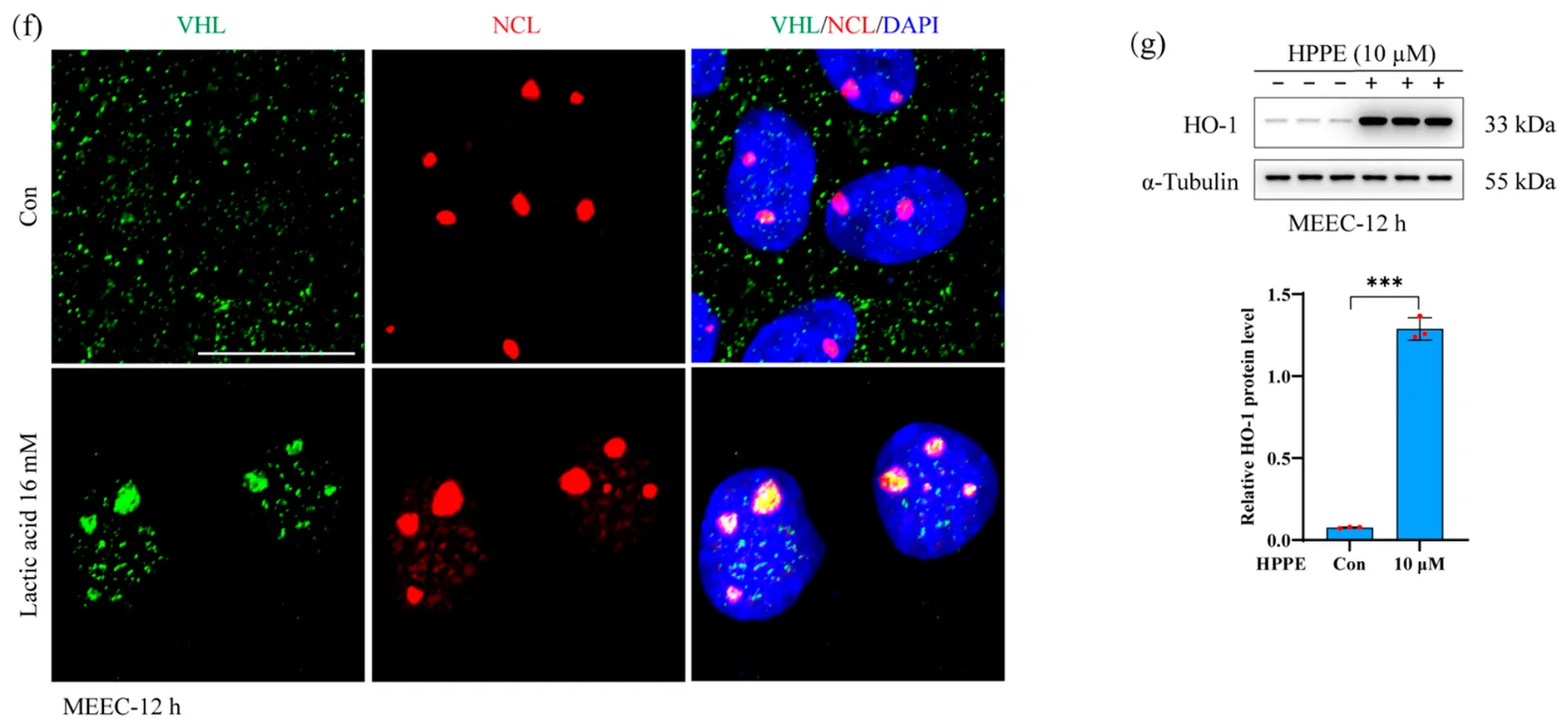
Lactic acid stabilizes HIF1α through PHD2/3 downregulation and VHL nucleolar sequestration, and suppresses BACH1 to drive uterine epithelial HO-1 expression. (**a**) Western blot analysis of HIF1α, HO-1, BACH1, BACH2, VHL, PHD2, and PHD3 protein levels after endometrial epithelial cells were treated with different concentrations of lactic acid for 12 h. (**b**) Immunofluorescence analysis of HIF1α, HO-1, and BACH1 in endometrial epithelial cells treated with 16 mM lactic acid for 12 h. Scale bar = 150 µm. (**c**) Immunofluorescence of HIF1α and HO-1 in epithelial organoids treated with 16 mM lactic acid for 12 h. Scale bar = 100 µm. (**d**) Western blot analysis and quantification of HIF1α, HO-1, BACH1, PHD2, PHD3, and VHL protein levels in ovariectomized (OVX) mouse uteri 3 h after intrauterine injection of 5 µL saline (control) or 5 µL of 1 mM lactic acid (*n* = 4 per group). (**e**) Immunofluorescence analysis of HIF1α, HO-1, BACH1, PHD2, and PHD3 in uterine tissue sections from OVX mice 3 h after intrauterine injection with 5 µL of 1 mM lactic acid (or saline as control). Scale bar = 50 µm. (**f**) Immunofluorescence analysis of VHL and NCL in endometrial epithelial cells treated with 16 mM lactic acid for 12 h. Scale bar = 150 µm. (**g**) Western blot analysis and quantification of HO-1 protein levels after endometrial epithelial cells were treated with 10 µM of HPPE for 12 h (*n* = 3 per group). Data were presented as mean ± SD. *: *p* < 0.05; **: *p* < 0.01; ***: *p* < 0.001, by two-tailed Student’s *t*-test.

In mEECs, lactic acid (8–16 mM) induced a concentration-dependent increase in HIF1α and HO-1 protein levels under normoxia, which was accompanied by reduced expression of PHD2 and PHD3 (Figure 4a). Immunofluorescence confirmed that 16 mM lactic acid enhanced nuclear HIF1α and cytoplasmic HO-1 signals in mEECs (Figure 4b). We observed the same coordinated regulation in uterine organoids, where confocal imaging revealed stronger nuclear HIF1α and cytoplasmic HO-1 after 12 h of exposure to 16 mM lactic acid compared with vehicle controls (Figure 4c). We next used an ovariectomized (OVX) model to confirm this regulation in vivo. Compared to controls, there were increased uterine HIF1α and HO-1, and decreased PHD2 and PHD3 3 h after intrauterine instillation of OVX mice with 5 µL of 1 mM lactic acid (Figure 4d). Immunofluorescence also showed that lactic acid enhanced HIF1α and HO-1 signals specifically in the epithelium, while reducing epithelial PHD2 and PHD3 immunofluorescence compared with saline controls (Figure 4e). These data indicate that lactic acid reduces HIF1α hydroxylation though PHD2 and PHD3, thereby interfering with its recognition by the degradation machinery.

Given that VHL targets HIF1α for degradation [38,70], we asked whether lactic acid regulates this process. Lactic acid also reduced VHL protein levels in mEECs and OVX uteri (Figure 4a,d). Immunofluorescence staining in mEECs showed that lactic acid promoted nucleolar sequestration of VHL, where VHL was co-localized with the nucleolar marker nucleolin (NCL) [71] (Figure 4f).

Together, the downregulation of PHD2 and PHD3, combined with VHL nucleolar sequestration, provides a dual mechanism by which lactic acid promotes robust HIF1α stabilization in the uterine epithelium. These data establish that lactic acid drives HO-1 expression by stabilizing HIF1α.

Given that BACH1 functions as an endogenous repressor of HO-1 [72,73], we asked whether lactic acid relieves this repression to induce HO-1. In mEECs, lactic acid (16 mM) suppressed BACH1 protein, whereas BACH2 showed no apparent change (Figure 4a). Immunofluorescence showed that 16 mM lactic acid reduced BACH1 fluorescence in mEECs (Figure 4b). In vivo, uterine BACH1 levels were decreased 3 h after intrauterine instillation of OVX mice with lactic acid (Figure 4d). Importantly, the hypoxia mimetic CoCl_2_ did not cause BACH1 downregulation (Figure 3b). Exposure to 3.5% O_2_ similarly had no effect on BACH1 levels (Figure 3c). These results indicate that BACH1 suppression is a lactic acid-specific event, rather than a general consequence of HIF1α activation.

Having established that lactic acid suppresses BACH1, we next asked whether heme also modulates BACH1. Heme is a key component of the HO-1-heme pathway [21]. After mEECs were treated with hemin (25 µM, 6 h), we observed a decrease in BACH1 protein levels and a concomitant increase in HO-1 expression (Figure S3a, Supporting Information). 5-Aminolevulinic acid (ALA) is a heme synthesis precursor [74]. Treatment of mEECs with ALA (1 mM, 12 h) caused reduced BACH1 and upregulated HO-1 (Figure S3b, Supporting Information). Thus, BACH1 is a common target of both lactic acid and heme.

To further test BACH1 regulation on *Hmox1* repression, we treated mEECs with the BACH1 inhibitor HPPE [75]. Pharmacological inhibition of BACH1 markedly upregulated HO-1 (Figure 4g), confirming that BACH1 functions as a critical brake.

### 3.5. Lactic Acid Mobilizes Heme and Upregulates HCAR2 in the Peri-Implantation Uterus

**Figure 5:**
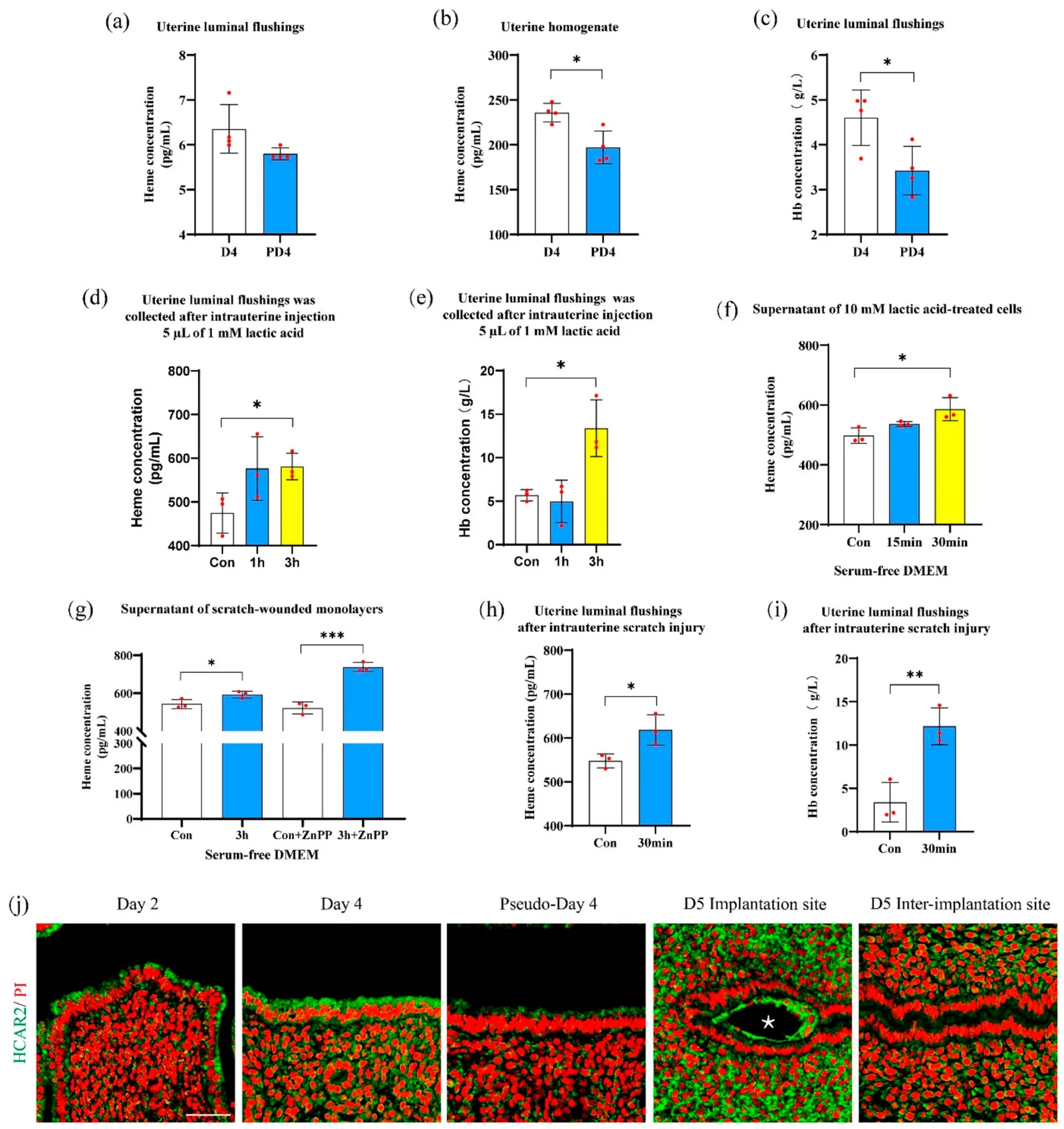
Effects of lactic acid on heme levels and immunofluorescence of uterine hemin receptor HCAR2. (**a**) Heme concentrations measured by ELISA in uterine luminal flushings from D4 and PD4 mice (*n* = 4 per group). (**b**) Heme concentrations measured by ELISA in uterine homogenates from D4 and PD4 mice (*n* = 4 per group). (**c**) Hb concentrations measured by ELISA in uterine luminal flushings from D4 and PD4 mice (*n* = 4 per group). (**d**) ELISA quantification of heme concentration in uterine luminal flushings from OVX mice 3 h after intrauterine of 5 µL saline or 5 µL of 1 mM lactic acid (*n* = 3 per group). (**e**) ELISA quantification of hemoglobin (Hb) levels in uterine luminal flushings from OVX mice 3 h after intrauterine injection of 5 µL saline or 5 µL of 1 mM lactic acid (*n* = 3 per group). (**f**) Heme levels in culture supernatants of mEECs treated with 10 mM lactic acid for 15 min and 30 min (*n* = 3 per group). (**g**) Heme levels in mEECs supernatants under the indicated conditions: untreated control, 3 h after scratch wounding, 3 h after treated with the HO-1 inhibitor ZnPP, and pretreated with ZnPP for 1 h and 3 h after scratch-wounding (*n* = 3 per group). (**h**) Heme levels in uterine luminal flushings 30 min after the uterine lumen of day 4 pseudopregnant mice was mechanically scratched. The unscratched contralateral horn was served as controls (*n* = 3 per group). (**i**) Hb levels in uterine luminal flushings 30 min after the uterine lumen of day 4 pseudopregnant mice was mechanically scratched. The unscratched contralateral horn was served as controls (*n* = 3 per group). (**j**) HCAR2 immunofluorescence in mouse uteri from D2, D4, PD4, and implantation site and non-implantation sites (NS) on D5. Scale bar = 50 µm. ☆ Embryo. Data were presented as mean ± SD. *: *p* < 0.05; **: *p* < 0.01; ***: *p* < 0.001, by two-tailed Student’s *t*-test.

Given that lactic acid induces uterine epithelial HO-1 and that HO-1 catalyzes the rate-limiting step of heme degradation [56], we asked whether lactic acid regulates heme metabolism and investigated how heme availability is controlled in the peri-implantation uterus. Heme concentrations in uterine luminal flushings of day 4 pregnant mice were higher compared with day 4 pseudopregnant uteri, but this difference did not reach statistical significance (Figure 5a). By contrast, heme levels in tissue homogenate were significantly elevated in day 4 pregnant uteri relative to day 4 pseudopregnant uteri (Figure 5b). Hemoglobin is the major storage form of heme [76]. We found that hemoglobin levels in uterine luminal flushings were significantly higher on day 4 of pregnancy than that on day 4 of pseudopregnancy (Figure 5c). Collectively, these data reveal a spatial distribution of heme that suggests mobilization by embryonic signals.

Because we previously identified lactic acid as a candidate embryo-derived regulator of uterine epithelial HO-1, we next tested whether lactic acid also mobilizes heme. Intrauterine injection of ovariectomized mice with lactic acid significantly increased heme concentrations in uterine luminal flushings within 3 h (Figure 5d). Hemoglobin levels were also elevated under the same conditions (Figure 5e). In mEECs, treatment with 10 mM lactic acid markedly increased heme levels in the culture supernatant (Figure 5f). Mechanical wounding of mEEC monolayers, which mimics the local tissue stress of implantation, similarly elevated heme in the supernatant (Figure 5g). Zinc protoporphyrin IX (ZnPP) is a competitive inhibitor of HO-1 [77]. Inhibition of HO-1 by ZnPP markedly elevated heme levels in the supernatant of wounded mEECs (Figure 5g). Consistent with this, mechanical scratching of the uterine lumen in day 4 pseudopregnant mice significantly elevated heme levels in uterine luminal flushings compared with uninjured uteri (Figure 5h). Similarly, hemoglobin levels in uterine luminal flushings were increased after mechanical scratching of day 4 pseudopregnant mice (Figure 5i). Together, these results identify lactic acid and implantation-associated tissue remodeling as key drivers of heme mobilization in the peri-implantation uterus.

Given the increased heme availability at the maternal-fetal interface, we examined the expression of HCAR2, a membrane receptor responsive to heme [78]. Immunofluorescence staining showed prominent HCAR2 expression in the luminal epithelium on day 4 of pregnancy, at significantly higher intensity than in pseudopregnant uteri (Figure 5j). These data identify lactic acid as a potent mobilizer of heme. This heme mobilization is accompanied by HCAR2 upregulation in the maternal epithelium, suggesting a potential paracrine circuit at the maternal-fetal interface.

### 3.6. HO-1-Mediated Heme Catabolism and Bilirubin Production Regulate Uterine Receptivity and Decidualization

**Figure 6:**
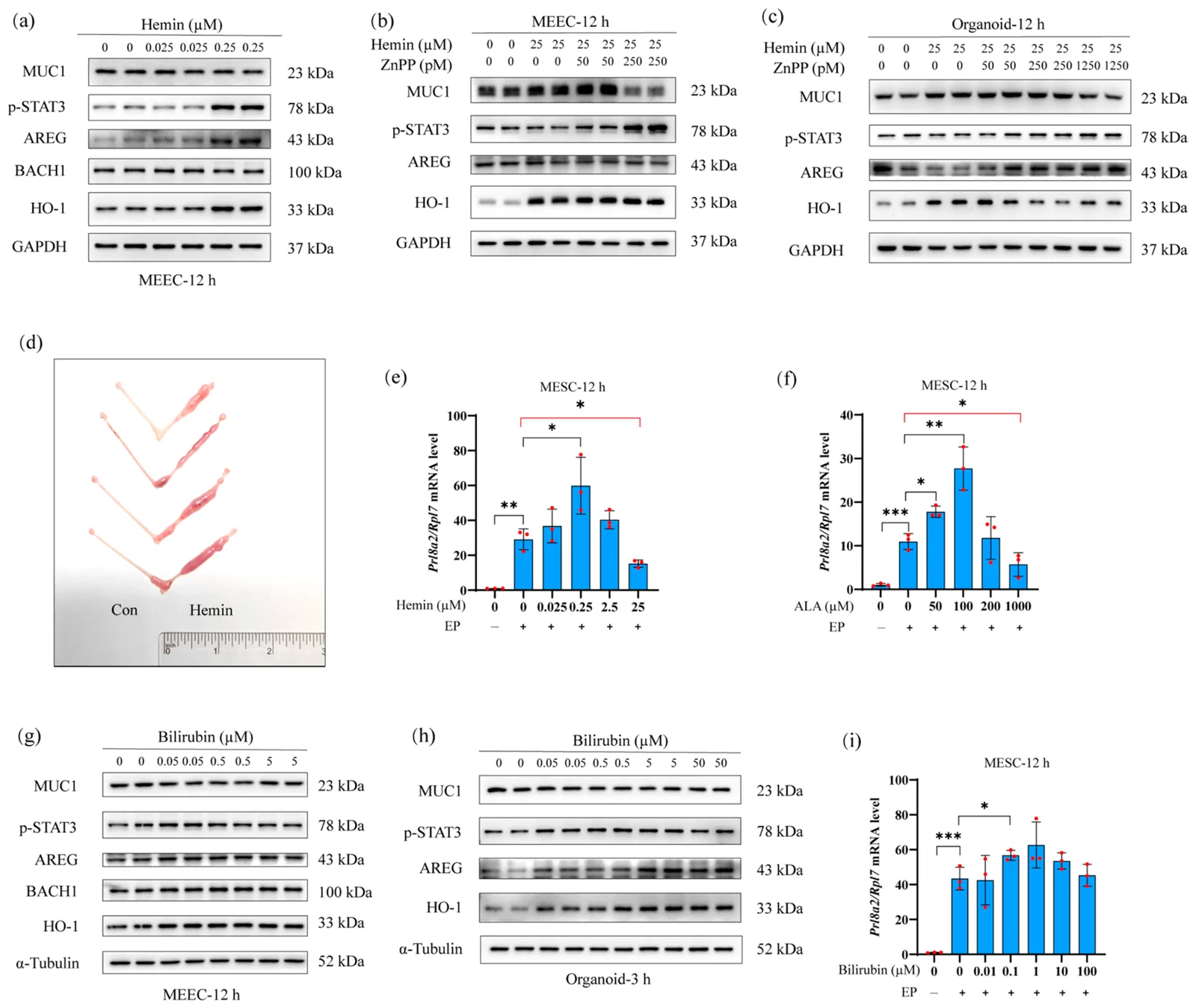
Heme and bilirubin regulation on uterine receptivity and decidualization. (**a**) Western blot analysis of MUC1, p-STAT3, AREG, BACH1, and HO-1 protein levels in mEECs treated with vehicle or different concentrations of hemin (0.025, 0.25 µM) for 12 h. (**b**) Western blot analysis of MUC1, p-STAT3, AREG, and HO-1 protein levels in mEECs treated with vehicle, hemin (25 µM), or hemin plus a low dose of the HO-1 inhibitor ZnPP (50, 250 pM) for 12 h. (**c**) Western blot analysis of MUC1, p-STAT3, AREG, and HO-1 in epithelial organoids treated with vehicle, hemin (25 µM), or hemin plus a low dose of the HO-1 inhibitor ZnPP (50, 250, 1250 pM) for 12 h. (**d**) The morphology of mouse uteri on day 8 of pseudopregnancy following unilateral intraluminal injection of hemin (10 mg/mL, 10 µL) into one uterine horn on day 4 of pseudopregnancy. The contralateral side received vehicle served as an internal control. The hemin-injected side showed a pronounced decidual response (*n* = 4 per group). (**e**) qPCR analysis on effects of hemin on *Prl8a2* mRNA levels under in vitro decidualization for 12 h (*n* = 3 per group). EP, treatment with estradiol-17β and progesterone for in vitro decidualization. (**f**) qPCR analysis on effects of ALA on *Prl8a2* mRNA levels under in vitro decidualization for 12 h (*n* = 3 per group). (**g**) Western blot analysis of MUC1, p-STAT3, AREG, BACH1, and HO-1 protein levels in mEECs treated with vehicle or different concentrations of bilirubin for 12 h. (**h**) Western blot analysis of MUC1, p-STAT3, AREG, BACH1, and HO-1 protein levels in epithelial organoids treated with vehicle or different concentrations of bilirubin for 3 h. (**i**) qPCR analysis on effects of bilirubin on *Prl8a2* mRNA levels under in vitro decidualization for 12 h (*n* = 3 per group). Data were presented as mean ± SD. *: *p* < 0.05; **: *p* < 0.01; ***: *p* < 0.001, by two-tailed Student’s *t*-test.

Given that heme is present in the peri-implantation uterus and its receptor HCAR2 is upregulated at the maternal-fetal interface, we next asked whether altered heme homeostasis influences endometrial receptivity and decidualization. As a pro-oxidant damage-associated molecular pattern (DAMP) released during tissue remodeling, heme can act as a sterile inflammatory signal [12,79]. We next examined the effects of hemin on epithelial receptivity markers in mEECs and organoids. In mEECs, treatment with a low concentration of hemin (0.25 µM, 12 h) increased p-STAT3 and AREG protein levels and reduced MUC1 expression (Figure 6a). By contrast, short-term exposure to a high concentration of hemin (25 µM, 6 h) in mEECs was sufficient to downregulate p-STAT3 and AREG and upregulate MUC1, indicating early disruption of epithelial homeostasis (Figure S3a, Supporting Information). Prolonged treatment with hemin (25 µM, 12 h) further exacerbated this dysfunction, as shown by persistent downregulation of p-STAT3 and AREG and upregulation of MUC1 (Figure 6b). This epithelial dysfunction was accompanied by excessive HO-1 upregulation. Pretreatment of mEECs with a picomolar concentration of ZnPP (250 pM, 1 h) before hemin (25 µM, 12 h), attenuated the downregulate of p-STAT3 and upregulation of MUC1, and also attenuated excessive HO-1 induction (Figure 6b). In epithelial organoids, ZnPP pretreatment (1250 pM, 1 h) followed by hemin (25 µM, 12 h) increased p-STAT3 and AREG levels, and reduced MUC1 expression, thereby attenuating hemin-induced epithelial dysfunction (Figure 6c). To test whether increasing endogenous heme synthesis would similarly disrupt epithelial homeostasis. We treated mEECs with ALA (1 mM) for 12 h. ALA reduced p-STAT3 and AREG protein levels and increased MUC1, confirming that excess heme production compromises epithelial receptivity (Figure S3b, Supporting Information).

Sterile inflammatory signals such as ATP and HMGB1 can induce decidualization [10,11]. We therefore asked whether hemin, acting as a potential sterile inflammatory signal, could also induce decidualization in vivo. Intrauterine injection of hemin induced extensive deciduoma formation in pseudopregnant uteri (Figure 6d), indicating that hemin can promote decidualization in vivo. We then tested the effects of hemin on stromal cell decidualization in vitro. Low-dose hemin (0.25 µM, 12 h) promoted decidualization, whereas high-dose hemin (25 µM, 12 h) suppressed this process (Figure 6e). Similarly, the heme synthesis precursor ALA enhanced decidualization at 50–100 µM (12 h) but inhibited decidualization at 1 mM (Figure 6f).

Because the protective effects of HO-1 are partly mediated by its downstream metabolite bilirubin [80], we next asked whether bilirubin directly modulates uterine receptivity and decidualization. In mEECs, treatment with bilirubin (0.05 µM) for 12 h increased p-STAT3 and AREG levels and reduced MUC1 expression (Figure 6g). In epithelial organoids, a higher concentration of bilirubin (0.5 µM) for 3 h similarly enhanced p-STAT3 and AREG and decreased MUC1 (Figure 6h). In primary stromal cells, bilirubin (0.1 µM, 12 h) promoted decidualization (Figure 6i). These results indicate that bilirubin enhances both epithelial receptivity and stromal decidualization.

In contrast to heme and lactic acid, bilirubin treatment did not alter BACH1 levels (Figure 6g), indicating that BACH1 suppression is specifically mediated by the upstream signals of lactic acid and heme. These findings show that HO-1-mediated heme degradation and bilirubin production influence uterine receptivity and decidualization.

### 3.7. Chronic Heme Excess Disrupts Uterine Receptivity and Impairs Embryo Implantation

**Figure 7:**
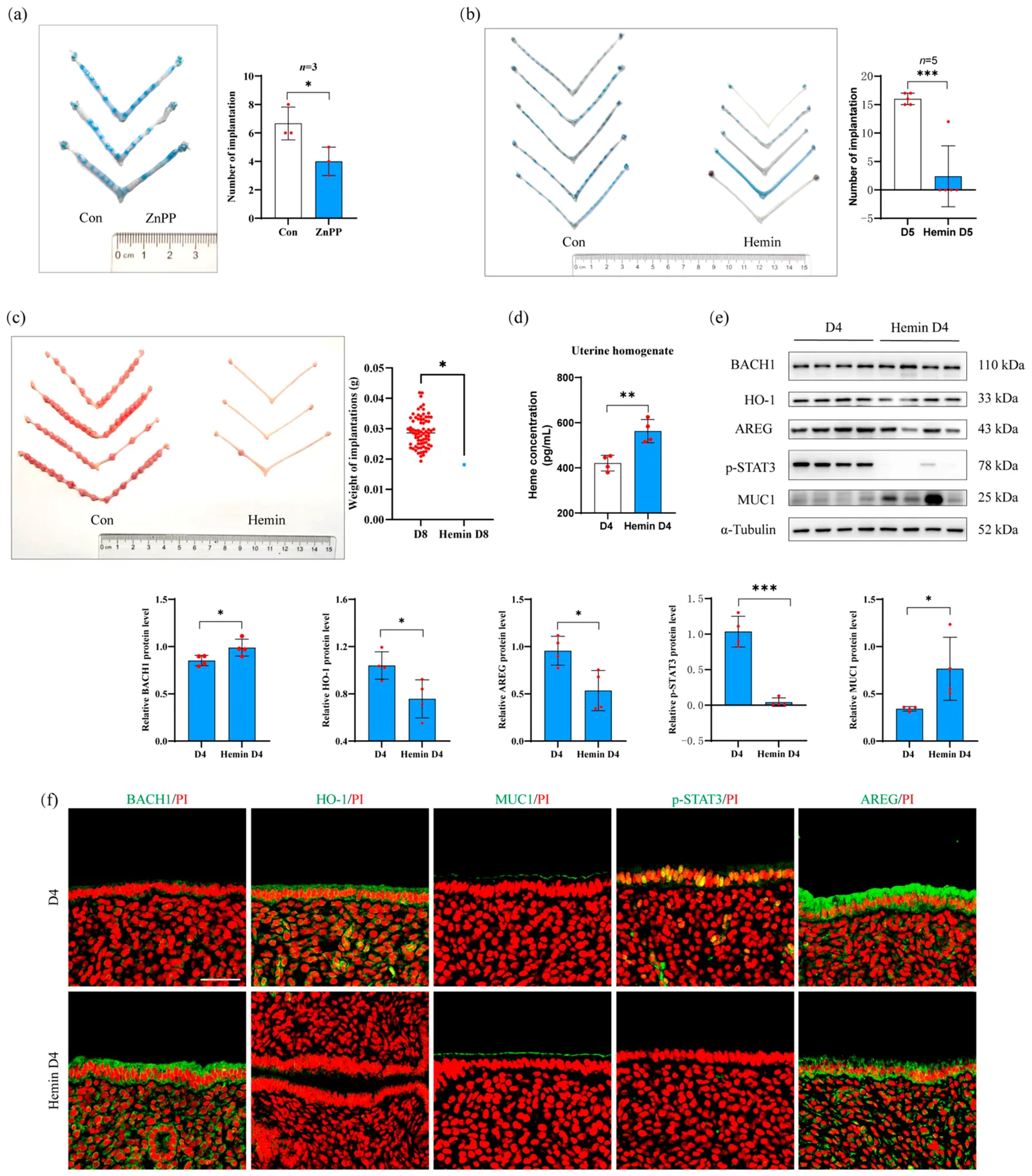
Effects of chronic dietary heme on uterine receptivity and embryo implantation after female mice were exposed to chronic hemin through oral gavage (0.2 mL per mouse, 10 mg Hemin/mL) for 4 weeks and mated with fertile males. (**a**) The morphology and number of implantation sites on day 5 of pregnancy after day 4 pregnant mice were intraluminal injected with 3 µL (3 mg/mL, per horn) of HO-1 inhibitor ZnPP (*n* = 3 per group). (**b**) The morphology and number of implantation sites on day 5 of pregnancy in control and heme-fed mice (*n* = 5 per group). (**c**) The uterine morphology and weight of implantation sites on day 8 of pregnancy in control and heme-fed mice (*n* = 3 per group). (**d**) Heme concentration in uterine homogenates measured by ELISA on day 4 of pregnancy (*n* = 4 per group). (**e**) Western blot analysis and quantification of BACH1, HO-1, p-STAT3, MUC1, and AREG in mouse uteri on day 4 of pregnancy in control and heme-fed mice (*n* = 4 per group). (**f**) Immunofluorescence analysis of BACH1, HO-1, p-STAT3, AREG, and MUC1 in mouse uteri on day 4 of pregnancy in control and heme-fed mice. Scale bar = 50 µm. Data were presented as mean ± SD. *: *p* < 0.05; **: *p* < 0.01; ***: *p* < 0.001, by two-tailed Student’s *t*-test.

Given the prominent expression of HO-1 in the peri-implantation uterine epithelium, we first asked whether HO-1 is required for embryo implantation. We locally inhibited HO-1 by intrauterine injection of the HO-1 inhibitor zinc protoporphyrin IX (ZnPP) [77]. Injection of ZnPP (3 µL per horn, 3 mg/mL) on day 4 of pregnancy significantly decreased the number of implantation sites on day 5 compared with the contralateral vehicle-treated horns (Figure 7a). These data indicate that HO-1 plays a critical role in embryo implantation.

Having established that HO-1 is essential for implantation, we next examined whether dysregulated heme homeostasis from dietary hemin compromises this process. We sought to mimic the sustained high heme intake associated with a red meat-rich diet, as previously described [81]. In our study, female mice were administered hemin by daily oral gavage (10 mg/mL, 0.2 mL per mouse) for 4 weeks before mating. This treatment significantly reduced the number of implantation sites on both days 5 and 8 of pregnancy, indicating severe early pregnancy failure (Figure 7b,c). Consistently, uterine heme levels were markedly elevated in hemin-treated mice compared with controls on day 4, confirming excessive uterine heme deposition (Figure 7d).

To investigate the molecular basis of this receptivity failure, we assessed markers of uterine receptivity in heme-treated and control mice on day 4 of pregnancy. Immunoblot analysis revealed that hemin treatment increased BACH1 and decreased HO-1 protein levels in the uterus compared with controls (Figure 7e). The epithelial receptivity markers p-STAT3 and AREG were strongly downregulated, whereas MUC1 was markedly upregulated (Figure 7e). These findings indicate that excessive heme disrupts the BACH1-HO-1 regulatory balance and locks the uterus in a non-receptive state. Moreover, immunofluorescence of uterine sections on day 4 of pregnancy revealed that hemin treatment increased BACH1 fluorescence intensity and decreased HO-1 intensity in the luminal epithelium (Figure 7f). Furthermore, p-STAT3 and AREG immunofluorescence intensity was markedly reduced in the luminal epithelium of hemin-treated mice compared with controls, whereas MUC1 signal was intensified (Figure 7f).

Collectively, these findings indicate that chronic heme excess disrupts the BACH1-HO-1 regulatory balance, resulting in loss of uterine receptivity and implantation failure.

## 4. Discussion

Successful embryo implantation relies on the precise spatiotemporal coordination of uterine receptivity and embryonic developmental competence [82]. Here, we identify a metabolic regulatory axis in which lactic acid regulates uterine epithelial receptivity and stromal decidualization through the HIF1α-HO-1-heme pathway in mice. Given that excessive dietary heme intake has been associated with an increased risk of colorectal cancer in mice and rats [15,16], we examined the impact of chronic heme exposure on implantation. Excess heme unexpectedly impairs uterine receptivity and causes implantation failure by downregulating HO-1 expression and disrupting uterine heme homeostasis. These findings establish that HO-1-mediated heme homeostasis is essential for early pregnancy, reveal a metabolic link between embryonic signals and maternal uterine adaptation, and identify a potential modifiable risk factor for implantation-related infertility.

The present study demonstrates that the implanting blastocyst selectively upregulates the stress-responsive isoform HO-1 in the luminal epithelium, while leaving the constitutive HO-2 unchanged. Our data further demonstrate that lactic acid, rather than other blastocyst-secreted factors such as CTSB or TNF [42,57,58,59], is the primary embryo-derived signal that stabilizes HIF1α in the luminal epithelium and thereby transcriptionally induces HO-1. Consistent with this, pregnant uteri exhibited elevated lactic acid levels and increased total LDH protein, accompanied by increased epithelial LDHA, lactic acid receptor GPR81, and V-ATPase subunits (ATP6V1A and ATP6V1B2). Enhanced LysoSensor Green fluorescence further verified embryo-induced luminal acidification. Collectively, these data establish lactic acid as the principal paracrine signal that induces epithelial HO-1 expression. The concurrent upregulation of GPR81 and V-ATPase subunits suggests a coordinated epithelial response to lactic acid. GPR81 may serve as a lactic acid sensor in mice and humans to initiate intracellular signaling [65], while V-ATPase-driven proton extrusion reinforces luminal acidification in mice, creating a positive feedback loop that amplifies the local lactic acid signal [53]. Notably, lactic acid has been reported to act through HIF1α stabilization in bovine mammary epithelial cells [83] and to upregulate HO-1 via histone lactylation and the HIF1α axis in human endometriosis cells [84]. These independent observations support the view that the lactic acid-HIF1α-HO-1 cascade represents a broadly utilized mechanism linking metabolic cues to cellular stress adaptation.

The uterine lumen is intrinsically acidic, consistent with earlier findings in the mouse uterus during the peri-implantation period [53]. In our study, the LDHA expression and detection of lactic acid in day 4 pseudopregnant mouse uteri confirm that the maternal endometrium has a basal capacity for lactic acid production. However, the acidic signal (LysoSensor Green DND-189 probe), LDHA fluorescence, and lactic acid content in uterine homogenates are higher in day 4 pregnant uteri than in day 4 pseudopregnant uteri. This indicates that the blastocyst amplifies uterine lactic acid output. This amplification arises from two synergistic mechanisms. First, the mouse blastocyst itself releases lactic acid, as shown by increased lactic acid concentration in culture medium after 48 h of embryo culture [60]. Second, embryonic signals upregulate uterine LDHA expression, thereby boosting maternal lactic acid production. We used exogenous lactic acid supplementation to mimic embryo-derived lactic acid. Therefore, our data suggest that lactic acid derived from mouse blastocysts should play a critical role during implantation.

Notably, the lactic acid-HIF1α-HO-1 axis is not embryo-exclusive. Pseudopregnant uteri in the absence of blastocysts still display detectable luminal acidification and basal HO-1 expression, indicating that the uterine epithelium possesses an intrinsic, embryo-independent capacity for HO-1 regulation in mice [53,85]. This basal activity is likely sustained by the physiological hypoxia inherent to the pre-implantation uterine microenvironment, a well-characterized driver of HIF1α accumulation and HO-1 induction across tissues. Our in vitro data confirm that both chemical (CoCl_2_) and physiological (3.5% O_2_) hypoxia are sufficient to stabilize HIF1α and upregulate HO-1 in mEECs. Building on this foundation, lactic acid further amplifies HO-1 expression through two complementary mechanisms. It reduces the abundance of PHD2 and PHD3 and promotes nucleolar sequestration of VHL in mammalian cells [38], thereby attenuating HIF1α hydroxylation and proteasomal degradation.

Concurrently, lactic acid diminishes the transcriptional repressor BACH1, thereby upregulating HO-1 expression in mouse models and human cancer cells [86,87]. BACH1 suppression is specific to lactic acid, as neither CoCl_2_ nor hypoxia reduces BACH1. Lactic acid thus engages a distinct regulatory layer beyond HIF1α stabilization, which removes a constitutive brake on *Hmox1* transcription. The combined effects on HIF1α and BACH1 explain why lactic acid induces HO-1 more potently than hypoxia alone. Stabilized HIF1α binds to hypoxia-response elements (HREs) in the *Hmox1* promoter, thereby activating its transcription. Intrauterine lactic acid administration in ovariectomized mice validated this lactic acid-HIF1α-HO-1 regulatory axis in vivo. Pharmacological inhibition of HO-1 with ZnPP markedly reduced implantation sites, confirming the importance of HO-1 in embryo implantation. Previous studies have reported that lactic acid can bind the catalytic domain of PHD2 [40] and that an acidic environment causes VHL sequestration in the nucleolus in mammalian cells [38], supporting the idea that lactic acid stabilizes HIF1α to induce HO-1. Although the HIF1α-HO-1 signaling axis has been studied mainly in human endometrial stromal cells [84], our work focuses on the uterine epithelium, because both HIF1α and HO-1 are predominantly localized to the luminal epithelium at day 5 implantation sites in mice, thereby refining the epithelial-specific role of this pathway in implantation.

Given that lactic acid induces epithelial HO-1, the rate-limiting enzyme in heme degradation [19]. We reasoned that this induction serves to maintain local heme homeostasis. We detected elevated heme concentrations in both uterine tissue homogenates and luminal fluid on day 4 of pregnancy, and further found that intrauterine injection of lactic acid into ovariectomized mice significantly increased heme levels in uterine luminal flushings within 3 h. These data identify lactic acid as a key driver of heme mobilization in the peri-implantation uterus. The concomitant upregulation of HCAR2 on the blastocyst suggests that the mobilized heme may also signal back to the embryo, establishing a bidirectional heme-mediated communication loop. In primary mEECs, lactic acid markedly elevated heme concentrations in the culture supernatant, establishing lactic acid as a key driver of heme mobilization in the peri-implantation uterus. Notably, HCAR2, which has been identified as a heme receptor in both humans and mice [78], was strongly expressed on the blastocyst at the implantation site on day 5, suggesting heme signaling may participate in embryo-uterine crosstalk. We therefore examined whether heme directly influence uterine receptivity and decidualization. Heme acts as a metabolic rheostat: low-dose hemin promotes an epithelial receptive state by increasing p-STAT3 and decreasing MUC1 in primary mEECs, and concurrently induces stromal cell decidualization, whereas High-dose hemin suppresses p-STAT3, upregulates MUC1, and inhibits decidualization.

Heme is an essential prosthetic group in diverse proteins and plays key roles in cellular physiology and metabolism. However, free heme released from cells and hemeproteins causes oxidative damage and inflammation [88]. Thus, maintaining heme homeostasis is essential for organismal health [89]. The concentration-dependent duality of heme positions it as a biological rheostat that translates graded heme availability into distinct cellular fates [90,91]. How does this rheostat operate at the maternal-fetal interface? Direct measurements of free heme in the uterine microenvironment are lacking. However, the regulatory labile heme pool in mammalian cells is about 433 ± 125 nM [92]. This is well below the approximately 1 µM threshold that separates signaling from toxicity [93]. Our 0.25 µM dose closely approximates this endogenous level and falls within the physiological range. Consistent with this, 0.25 µM hemin induces HO-1 expression and promotes epithelial receptivity and stromal decidualization, which are hallmarks of adaptive heme signaling rather than stress. However, heme level in murine uterine lavage fluid is approximately 2–5 µM and decreases to about 2 µM during the receptive phase, coinciding with peak HO-1 expression [85]. Although this reflects total rather than free heme, the decline supports reduced heme availability in the uterine microenvironment during embryo implantation. In contrast, pathological free-heme elevations occur in endometriosis peritoneal fluid (14.22 µM) [94]. Plasma labile heme is elevated in hemolytic diseases (2–50 µM) [95], and severe hemoglobinopathies (50–280 µM) [96]. Our 25 µM dose is 25-fold above the 1 µM threshold, exceeding the protective HO-1 induction range observed up to 20 µM [97]. However, 25 µM hemin surpasses this adaptive capacity and triggers ROS accumulation and lipid peroxidation as shown in HT22 neurons [98]. The suppression of decidualization at 25 µM therefore reflects heme-induced cellular stress. This stress arises when heme clearance capacity is exceeded, representing a pathological consequence rather than disruption of physiological signaling. Collectively, these data support a biphasic rheostat model at the maternal-fetal interface. Low heme (0.25 µM) supports endometrial adaptation, whereas high heme (25 µM) induces stress and impairs implantation.

HO-1-mediated heme degradation produces bilirubin, which promotes epithelial receptivity and stromal decidualization at low concentrations, indicating that the protective effects of HO-1 are mediated in part by this downstream metabolite [25]. Furthermore, hemin also as a sterile inflammatory mediator, inducing deciduoma formation in pseudopregnant uteri. These observations are consistent with clinical evidence that human endometrium possesses a complete heme metabolic machinery [99] and that excess free heme drives uterine pathologies through metabolic reprogramming and immune microenvironment remodeling in humans [100]. Collectively, these findings highlight that precisely regulated heme homeostasis is indispensable for uterine function, and its disruption links implantation failure to broader endometrial pathophysiology.

Given the link between dietary heme from red meat and colorectal cancer risk in mouse models [15,16], we examined whether chronic heme excess similarly disrupts uterine function in a dietary exposure model. Mice that received sustained heme gavage for one month before mating exhibited significantly fewer implantation sites. This was accompanied by elevated uterine heme levels, accumulation of the transcriptional repressor BACH1, and consequent suppression of HO-1 expression. At the molecular level, chronic heme excess reduced p-STAT3 and upregulated MUC1, indicating a disrupted epithelial receptive state and leading to implantation failure. These data demonstrate that heme accumulation in the uterus exceeds the HO-1-mediated clearance capacity, leading to BACH1 accumulation and impaired uterine receptivity. These findings extend the heme rheostat model to dietary heme excess and demonstrate that chronic disruption of heme homeostasis compromises uterine receptivity, identifying dietary heme as a preventable risk factor for implantation failure.

Our data reveal that lactic acid acts through a HIF1α-HO-1-heme axis to regulate implantation, and its disruption leads to implantation failure, highlighting HO-1 as a potential therapeutic target for implantation disorders. Beyond its rate-limiting role in heme degradation, HO-1 functions as a crucial antioxidant enzyme [101]. Its products biliverdin, carbon monoxide and free iron directly confer antioxidant defense against oxidative stress, a defining feature of the peri-implantation environment in mice [85]. Notably, HO-1 has been implicated in the suppression of alloimmune rejection by promoting T cell apoptosis in organ transplantation in mice [102]. Given that the embryo constitutes a semi-allogeneic graft, the prominent localization of HO-1 at the luminal epithelium suggests that its robust peri-implantation induction promotes maternal immune tolerance by restraining inflammation and preventing rejection, as demonstrated in mouse models of transplantation tolerance [103]. Future studies exploring whether pharmacological HO-1 induction or bilirubin supplementation can rescue implantation under metabolic stress conditions such as chronic heme exposure would further establish the therapeutic potential of this pathway. This suggests that robust epithelial HO-1 induction around implantation may have additional immunological roles.

We acknowledge two main limitations. First, our findings are derived from mouse models. Whether this axis operates in humans remains unknown. Second, some experiments have limited sample sizes (*n* = 3 biological replicates), which may not fully capture inter-individual variability. Future studies with larger sample size and human endometrial samples are warranted to validate the translational relevance of the heme-HO-1 axis during embryo implantation.

Taken together, our results establish a metabolic axis through which embryonic signals coordinate uterine adaptation during embryo implantation. Blastocyst-derived lactic acid drives HO-1 expression in the uterine luminal epithelium. Mechanistically, lactic acid stabilizes HIF1α through PHD2/3 downregulation and VHL nucleolar sequestration, while concurrently relieving BACH1-mediated repression of HO-1. This HIF1α-HO-1 axis is activated by embryo-derived lactic acid and physiological hypoxia. HO-1-mediated heme catabolism produces bilirubin, which promotes epithelial receptivity and decidualization. Low-dose hemin similarly promotes these processes, whereas high-dose hemin or chronic dietary heme impairs implantation. Notably, hemin acts as a metabolic rheostat with therapeutic and dietary implications. Collectively, these data identify an embryo-initiated metabolic checkpoint that safeguards uterine heme homeostasis and highlight that while the uterus provides a basal lactic acid contribution, the blastocyst is the primary driver of this signaling cascade.

## Figures and Tables

**Table 1:** qPCR Primer Sequences.

Genes	Species	Sequence (5′-3′)	Accession Number
*Rpl7*	Mouse	GCAGATGTACCGCACTGAGATTC	NM_011291.5
		ACCTTTGGGCTTACTCCATTGATA	
*Prl8a2*	Mouse	AGCCAGAAATCACTGCCACT	NM_010088
		TGATCCATGCACCCATAAAA	

Table notes: Every experiment was carried out at least three times.

## Data Availability

All data in this article are available.

## Supplementary Materials

The following supporting information can be downloaded at: https://www.mdpi.com/article/10.3390/antiox15091114/s1, Figure S1: TNF and CTSB failed to induce HO-1 in primary mouse endometrial epithelial cells; Figure S2: Expression and localization of HIF1α in mouse uterine tissue; Figure S3: Hemin and ALA disrupt the receptive state of primary mouse endometrial epithelial cells.
