# Supplementary material for: Blastocyst-Derived Lactic Acid Regulates Uterine Epithelial Receptivity and Stromal Decidualization via the HIF1α-HO-1-Heme Metabolic Axis
Source: Antioxidants (Basel). 2026 Sep 4;15(9):1114. doi: 10.3390/antiox15091114 (PMC13603621; doi:10.3390/antiox15091114)
Supplement: Supplementary file 1 [file antioxidants-15-01114-s001.zip › antioxidants-4507711-supplementary.pdf]

antioxidants

MDPI

Type of the Paper

Blastocyst-derived Lactic Acid Regulates Uterine Epithelial Receptivity and Stromal Decidualization via the HIF1α-HO-1-Heme Metabolic Axis

Wen-Xu Yao [1], Yao-Dan Ma [1], Shi-Yao Ding [1], Jian Lu [1], Hao-Lan Tang [1], Zeng-Ming Yang [1,*]

1 Key Laboratory of Animal Genetics, Breeding and Reproduction in the Plateau Mountain Region, College of Animal Science, Guizhou University, Guiyang, 550025, China.

* Corresponding Author. Email: yangzm@gzu.edu.cn

Supplementary information

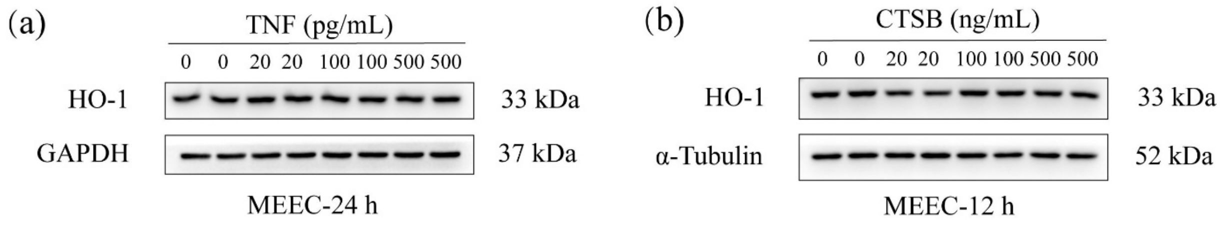

Figure S1. TNF and CTSB failed to induce HO-1 in primary mouse endometrial epithelial cells. (a) Western blot of HO-1 protein levels after endometrial epithelial cells were treated with different concentrations of TNF for 24 h. (b) Western blot of HO-1 protein levels after endometrial epithelial cells were treated with different concentrations of CTSB for 12 h.

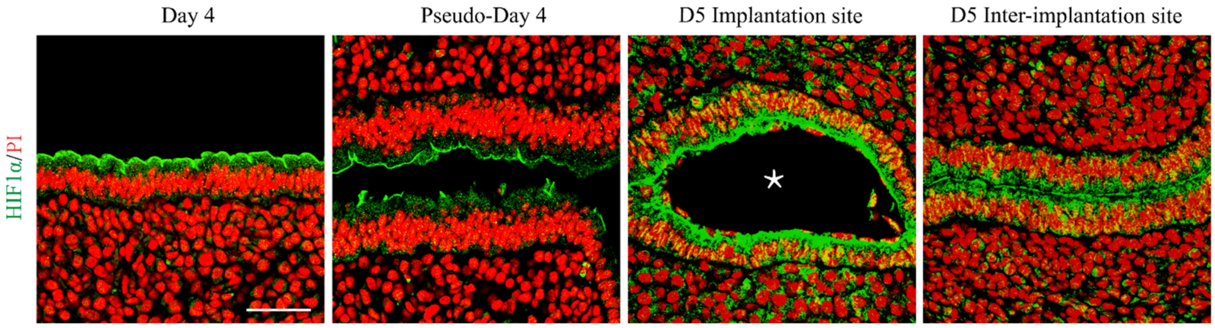

Figure S2. Expression and localization of HIF1α in mouse uterine tissue. Immunofluorescence of HIF1α in mouse uteri on day 4 of pregnancy and day 4 of pseudopregnancy, at implantation sites and inter-implantation sites on day 5. Scale bar = 50 μm. ☆ Embryo.

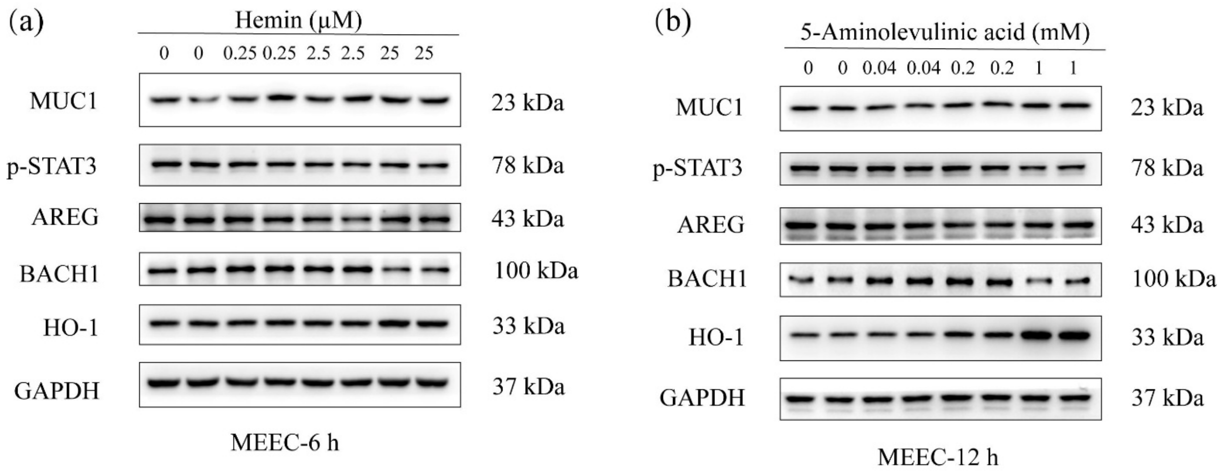

Figure S3. Hemin and ALA disrupt the receptive state of primary mouse endometrial epithelial cells. (a) Western blot analysis and quantification of MUC1, p-STAT3, AREG, BACH1, and HO-1 protein levels after endometrial epithelial cells were treated with different concentrations of Hemin for 6 h. (b) Western blot analysis and quantification of MUC1, p-STAT3, AREG, BACH1, and HO-1 protein levels after endometrial epithelial cells were treated with different concentrations of ALA for 12 h.

Disclaimer/Publisher's Note: The statements, opinions and data contained in all publications are solely those of the individual author(s) and contributor(s) and not of MDPI and/or the editor(s). MDPI and/or the editor(s) disclaim responsibility for any injury to people or property resulting from any ideas, methods, instructions or products referred to in the content.
